# A network model of Italy shows that intermittent regional strategies can alleviate the COVID-19 epidemic

**DOI:** 10.1038/s41467-020-18827-5

**Published:** 2020-10-09

**Authors:** Fabio Della Rossa, Davide Salzano, Anna Di Meglio, Francesco De Lellis, Marco Coraggio, Carmela Calabrese, Agostino Guarino, Ricardo Cardona-Rivera, Pietro De Lellis, Davide Liuzza, Francesco Lo Iudice, Giovanni Russo, Mario di Bernardo

**Affiliations:** 1 Department of Electronic, Information and Biomedical Engineering, Politecnico di Milano, Milan, Italy; 2 Department of Electrical Engineering and Information Technology, University of Naples Federico II, Naples, Italy; 3 ENEA, Fusion and Nuclear Safety Department, Frascati, Rome Italy; 4 Department of Information and Electrical Engineering and Applied Mathematics, University of Salerno, Fisciano, Italy

**Keywords:** Computational models, Viral infection, Applied mathematics

## Abstract

The COVID-19 epidemic hit Italy particularly hard, yielding the implementation of strict national lockdown rules. Previous modelling studies at the national level overlooked the fact that Italy is divided into administrative regions which can independently oversee their own share of the Italian National Health Service. Here, we show that heterogeneity between regions is essential to understand the spread of the epidemic and to design effective strategies to control the disease. We model Italy as a network of regions and parameterize the model of each region on real data spanning over two months from the initial outbreak. We confirm the effectiveness at the regional level of the national lockdown strategy and propose coordinated regional interventions to prevent future national lockdowns, while avoiding saturation of the regional health systems and mitigating impact on costs. Our study and methodology can be easily extended to other levels of granularity to support policy- and decision-makers.

## Abstract (web summary)

An ongoing global debate concerns effective and sustainable lockdown release strategies in the current pandemic. Here, the authors implement a network model at healthcare-relevant spatial scale to show that coordinated local strategies can be effective in containing further resurgence of the disease.

## Introduction

Regionalism is an integral part of the Italian constitution. Each of Italy’s twenty administrative regions is independent on Health and oversees its own share of the Italian National Health service. The regional presidents and their councils can independently take their own actions, strengthening or, at times, weakening national containment rules. Previous studies have modelled the spread of the epidemics and its evolution in the country at the national level^1–5^, and some have looked at the effects of different types of containment and mitigation strategies^6–11^. Limited work^12–21^ has taken into account the spatial dynamics of the epidemic but, to the best of our knowledge, no previous paper in the literature has explicitly taken into consideration the pseudo-federalist nature of the Italian Republic and its strong regional heterogeneity when it comes to health matters, hospital capacity, economic costs of a lockdown and the presence of inter-regional people’s flows.

In this study, we investigate the whole of the country as a network of regions, each modelled with different parameters. The goal is to identify if and when measures taken by the Italian government had an effect at both the national, but most importantly, at the regional level. Also, we want to uncover the effects on the epidemic spread of regional heterogeneity and inter-regional flows of people and use control theoretic tools to propose and assess differentiated interventions at the regional level to reopen the country and avoid future recurrent epidemic outbreaks.

**Fig. 1:**
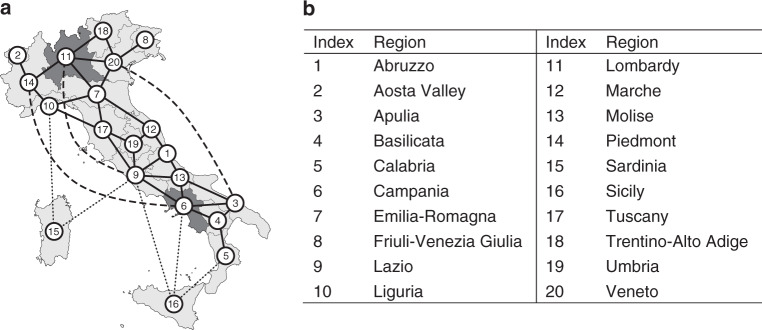
Schematic diagram of the network-model structure and representative regional parameters. **a** Representative graph of the network-model structure used in the paper. Only a subset of all links is shown for the sake of clarity (the complete graphs are depicted in Supplementary Fig. 8). Solid lines represent proximity links, dashed lines long-distance transportation routes (planes and trains), dotted lines show major ferry routes between insular regions and the Italian mainland. **b** Table of the Italian region names and their labels in the graph.

As aggregate models of the COVID-19 epidemic cannot capture these effects, to carry out our study we derived and parameterized from real data a network model of the epidemic in the country (see Fig. 1a), where each of the 20 regions is a node and the links model both proximity flows and long-distance transportation routes (ferries, trains and planes). The model is first shown to possess the right level of granularity and complexity to capture the crucial elements needed to correctly predict and reproduce the available data. Then, it is used to design and test differentiated feedback interventions at the regional level to alleviate the epidemic impact.

Using the model and an ad hoc algorithm to parameterize it from real data, we evaluate the effectiveness of the national lockdown strategy implemented by the Italian government providing evidence of its efficacy across regions. Also, we show that inter-regional fluxes must be carefully controlled as they can have dramatic effects on recurrent epidemic waves. Finally, we convincingly show that regional feedback interventions, where each of the twenty regions strengthens or weakens local mitigating actions (social distancing, inflow/outflow control) as a function of the saturation of their hospital capacity, can be beneficial in mitigating possible outbreaks and in avoiding recurrent epidemic waves while reducing the costs of a nationwide lockdown.

## Results

### Model formulation and fitting procedure

To capture the regional diversity of the response to the epidemic in Italy, we derive a network model where each node represents a different region and links capture fluxes of people traveling among the regions (see Fig. 1a). Using a data-driven compartmental modelling approach, a set of ODEs is obtained describing the dynamics of six different compartments in each region (Susceptibles, Infected, Hospitalized, Quarantined, Deceased and Recovered); data analysis being used (see Methods) to define flows among compartments. The resulting model is then parameterized using a predictor-corrector algorithm applied to both a national aggregate model and to each of the twenty regional models, identifying the time points at which parameter values present significant changes. Soft constraints are used to enforce continuity of the trajectory between different time windows and avoid parameters changing too abruptly (see Methods and Supplementary Notes for further details). Estimating all the parameters in each region allows us to fit the available data and to describe the different regional situations and the diverse impact that regional policies had on the epidemic spread in each of the Italian regions.

As further explained in the Methods and Supplementary Information, we fit the model parameters to the official data for the COVID-19 epidemic^22^, as collected by the Dipartimento della Protezione Civile—Presidenza del Consiglio dei Ministri (the Italian Civil Protection Agency). Also, publicly available mobility data is used to estimate inter-regional fluxes and data on the number of ICU beds^8,9^ to evaluate the capacities of regional health services. To assess the economic costs of national and regional lockdowns we use official data and estimates from Italian governmental agencies^23–26^. Further details on the input data and the official repositories they were obtained from can be found at https://github.com/diBernardoGroup/Network-model-of-the-COVID-19.

### Regional effects of the national lockdown

Our approach successfully uncovers the regional effects of the national lockdown measures set in place by the Italian government initially in two northern regions (Lombardy and Veneto from the February 27, 2020), and then nationally from March 8, 2020 till May 4, 2020. We observe that notable parameter changes, detected automatically by our parameterization procedure (see Methods), occur as an effect of such measures with a certain degree of homogeneity across all regions (see Supplementary Fig. 13 and Supplementary Table 4 showing the changes in the social distancing parameter *ρ*_*i*_ over the period of interest). This confirms the effectiveness across the country of the strict social distancing rules implemented at the national level as also noted in previous work^1,2,14^ modelling the country as a whole.

The representative examples of two regions, Lombardy in the North and Campania in the South, highlighted in Fig. 1, show that the model correctly captures the effect of such measures in both the regions, see Table 1. The model also captures the effect of the flow of people that travelled from North to South when the national lockdown measures were first announced on March 8, 2020. As shown in Table 1, the estimated number of infected predicted by the model for the Campania region in the time window March 19–March 30, 2020 is detected to suddenly increase at the beginning of the next time window. This can be explained as a possible effect of the movement of people from North to South that occurred around 15 days before. Also, data analysis shows that the mortality rate varies as a function of the level of occupancy of the hospital beds in each region (see Supplementary Fig. 14 and Supplementary Notes for further details).

**Table 1:** Estimated parameter values for Campania in the South (region no. 6) and Lombardy in the North (region no. 11), where the initial outbreak occurred.

Region	Breakpoint	*ρ*_*i*_	*α*_*i*_	*ψ*_*i*_	*κ*_*i*_^*H*^	*κ*_*i*_^*Q*^	*η*_*i*_^*Q*^	*η*_*i*_^*H*^	*ζ*_*i*_	*I*_0_	*I*_*f*_	*R*_0,*i*_
Campania	19/3/20	0.467	0.014	0.064	0.000	0.100	0.018	0.000	0.022	1231	1816	1.26
	30/3/20	0.221	0.067	0.019	0.006	0.040	0.018	0.000	0.011	2231	234	0.57
Lombardy	27/2/20	0.727	0.009	0.092	0.000	0.040	0.010	0.053	0.033	1799	28900	1.69
	19/3/20	0.303	0.018	0.056	0.000	0.027	0.010	0.029	0.024	28900	6731	0.84

These regions are highlighted in a darker colour in Fig. 1. Here, *I*_0_ is the number of infected estimated in the region at the beginning of each time window, while *I*_*f*_ is the number of infected at the end of each time window estimated by running the model (14)–(16), given the set of identified parameters and the initial condition on the infected *I*_0_. The first breakpoint is the date when 10 deaths and 10 recovered were first reported in the region and the analysis started. The second breakpoint is the end of the first window and the start of the second window (ending on May 3, 2020).

### Regional heterogeneity counts

**Fig. 2:**
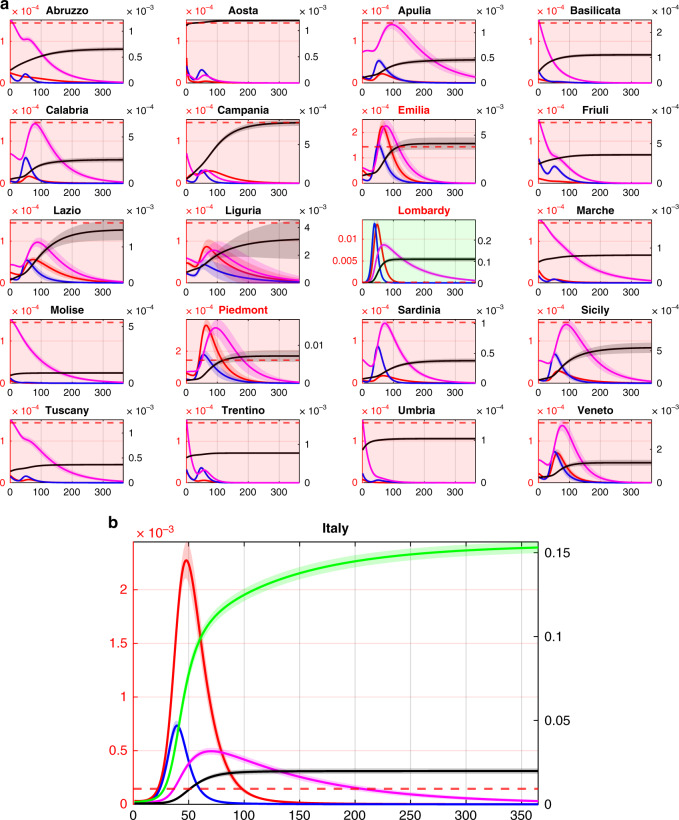
Only one region relaxes its lockdown. Double scale plots of the **a** regional and **b** national dynamics in the case where only one region (Lombardy in Northern Italy) relaxes its containment measures at time 0, while inter-regional fluxes are set to the level they reached during the lockdown. (Regional dynamics when the fluxes between regions are set to their prelockdown level are shown in Supplementary Fig. 1 showing even more dramatic scenarios.) The scale on the left vertical axis (in red) applies to the fraction of hospitalized requiring ICU (red solid line) and the ICU beds capacity threshold ($T_i^H{\mathrm{/}}N_i$, dashed red line). The scale on the right vertical axis (in black) applies to the infected (blue), quarantined (magenta), recovered (green) and deceased (black). The time scale, on the horizontal axis, is given in days. Panels of regions adopting a lockdown are shaded in red while those of regions relaxing social containment measures are shaded in green. Results are averaged over 10,000 simulations with parameters sampled using a Latin Hypercube technique (see Methods) around their nominal values set as those estimated in the last time window for each region as reported in Supplementary Table 4. Shaded bands correspond to twice the standard deviation. The regions identified with a red label are those where the total hospital capacity is saturated.

After confirming the predictive and descriptive ability of the proposed model, we investigated the influence of the regional heterogeneity on the onset of an epidemic outbreak and the occurrence of possible recurrent epidemic waves. To this aim we set the model with parameters capturing the situation in each region on May 3, 2020, when the effects of the national lockdown were fully in place, and simulated the scenario where just one of the twenty regions (e.g., Lombardy in the North of Italy) fully relaxes its lockdown. As reported in Fig. 2, we found that a primary outbreak in that region would quickly propagate causing secondary recurrent outbreaks in other regions including Emilia-Romagna and Piedmont. At the national level this would cause the onset of a second epidemic wave that, if not contained, would end up afflicting more than 25% of the entire population. An even more dramatic scenario would emerge if inter-regional flows were concurrently restored to their prelockdown levels (see Supplementary Fig. 1) or all regions were to relax their current restrictions concurrently (see Supplementary Fig. 2).

### Feedback regional interventions can be beneficial

**Fig. 3:**
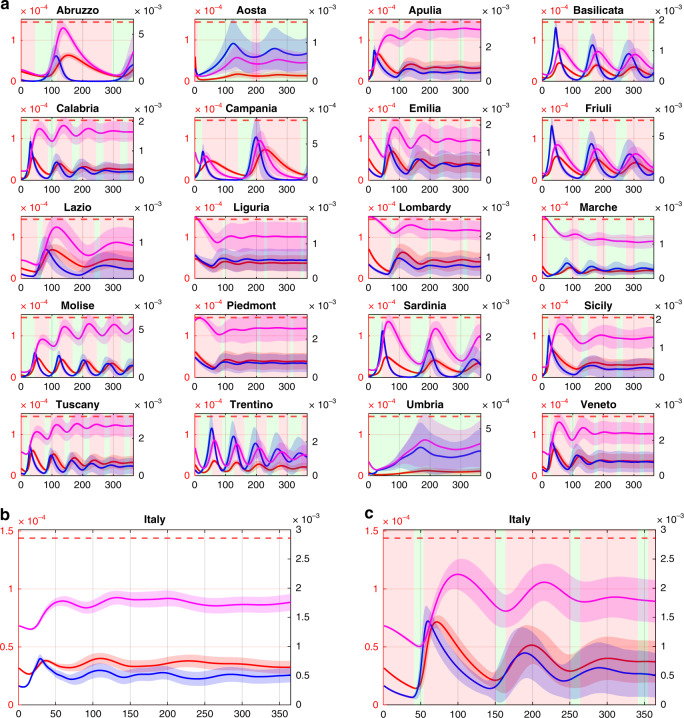
Intermittent regional measures. **a** Each of the 20 panels shows the evolution in a different region of the fraction in the population of infected (blue), quarantined (magenta) and hospitalized requiring ICUs (red) averaged over 10,000 simulations with parameters sampled using a Latin Hypercube technique (see Methods) around their nominal values set as those estimated in the last time window for each region as reported in Supplementary Table 4. Shaded bands correspond to twice the standard deviation. Dashed red lines represent the fraction of the population that can be treated in ICU ($T_i^H{\mathrm{/}}N_i$). Regions adopt lockdown measures in the time windows shaded in red while relax them in those shaded in green. During a regional lockdown, fluxes in/out of the region are set to their minimum level. **b** National evolution of the fraction in the population of infected (blue), quarantined (magenta) and hospitalized requiring ICUs (red) obtained by summing those in each of the 20 regions adopting intermittent regional measures. **c** National evolution of the fraction in the population of infected (blue), quarantined (magenta) and hospitalized requiring ICUs (red) when an intermittent national lockdown is enforced with all regions shutting down when the total number of occupied ICU beds at the national level exceed 20%, reopening when it goes back below 10%. Regional dynamics corresponding to this scenario are shown in Supplementary Fig. 4. All plots are shown with a double scale. The scale on the left vertical axis (in red) applies to the hospitalized requiring ICU and the ICU beds capacity threshold, while the right vertical axis (in black) applies to the infected and quarantined. The time scale, on the horizontal axis, is given in days.

**Fig. 4:**
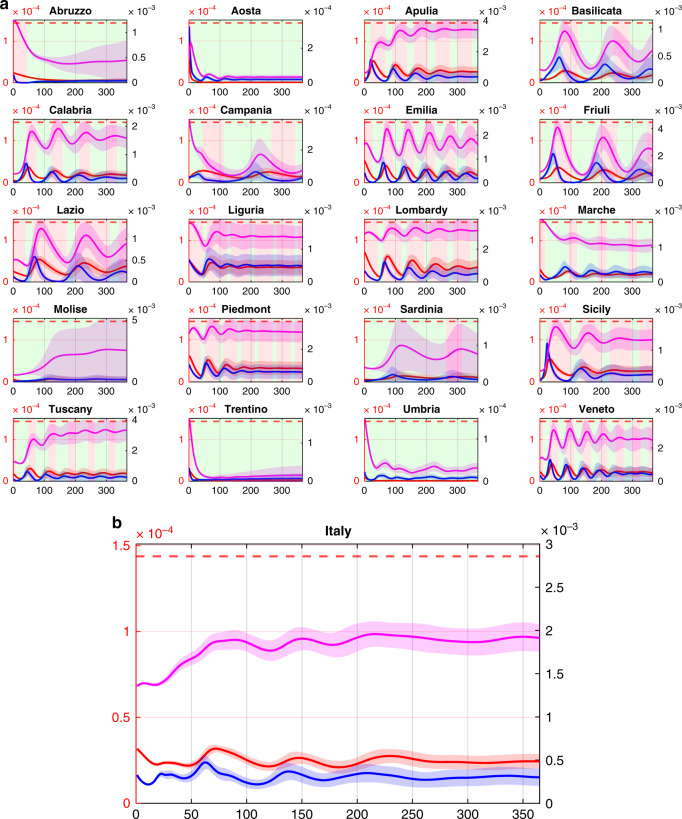
Intermittent regional measures with increased COVID-19 testing capacity. **a** Each of the 20 panels shows in a double scale plot the evolution in the region named above the panel of the fraction in the population of infected (blue), quarantined (magenta) and hospitalized requiring ICUs (red) averaged over 10,000 simulations with parameters sampled using a Latin Hypercube technique (see Methods) around their nominal values set as those estimated in the last time window for each region as reported in Supplementary Table 4. Shaded bands correspond to twice the standard deviation. Dashed red lines represent the fraction of the population that can be treated in ICU ($T_i^H{\mathrm{/}}N_i$). Regions adopt lockdown measures in the time windows shaded in red while relax them in those shaded in green. During a regional lockdown, fluxes in/out of the region are set to their minimum level. Regional COVID-19 testing capacities are assumed to be increased by a factor 2.5 (see Methods) with respect to their current values. **b** National evolution of the fraction of infected (blue), quarantined (magenta) and hospitalized requiring ICUs (red) obtained by summing those in each of the 20 regions adopting intermittent regional measures. All plots are shown with a double scale. The scale on the left vertical axis (in red) applies to the hospitalized requiring ICU and the ICU beds capacity threshold, while the right vertical axis (in black) applies to the infected and quarantined subjects.

A crucial open problem is to support decision-makers in determining what form of interventions might be beneficial to avoid the onset of future outbreaks while mitigating the cost of Draconian interventions at the national level. To this aim we compared the effects of national measures (e.g., general lockdown) against those of a regional feedback strategy, where social distancing measures are put in place or relaxed independently by each region according to the ratio between hospitalized individuals and the total capacity of the health system in that region. In particular, we assume that each region implements a stricter lockdown when such a ratio becomes greater than or equal to 20% and relaxes the social distancing rules when it is below 10% (see Methods for further details). Figure 3 confirms that a differentiated strategy among the regions (Fig. 3b) is as effective as a national lockdown in avoiding future waves of the epidemic (Fig. 3c and Supplementary Fig. 4). At the same time, an intermittent regional strategy guarantees that no region exceeds its own hospitals’ capacity and yields a lower economic cost for the country (Table 2 and Supplementary Table 1), since regional economies can be restarted and remain open for a much longer time. This advantage becomes even more apparent when regions concurrently increase their testing capacity as shown in Fig. 4 and reported in Table 2.

**Table 2:** Comparison of each of the simulated scenarios.

Simulation	Total cases	Total deaths	Maximum hospitalized	Days over hospital’s capacity (nation)	Regions over hospital’s capacity	Economic cost [M€]
All regions but Lombardy are locked down (Fig. 2)	10,550,000 ± 146,084	1,196,063 ± 97,122	137,640 ± 10,249	75.8 ± 2.7	3	503,355 ± 0
Intermittent regional measures (Fig. 3a, b)	1,986,601 ± 76,184	173,637 ± 3911	2801 ± 170	0 ± 0	0	509,142 ± 6606
Intermittent national measure (Fig. 3c, S4)	2,162,539 ± 194,929	205,261 ± 10,854	4481 ± 277	0 ± 0	3	562,373 ± 12,809
Intermittent regional measures with increased testing (Fig. 4)	1,590,459 ± 69,118	128,644 ± 2690	2057 ± 102	0 ± 0	0	366,514 ± 12,258

Metrics to evaluate the impact over 1 year of each of the simulated scenarios are reported showing the effectiveness of the intermittent regional measures shown in Figs. 3 and 4 in avoiding any saturation of the regional health systems while mitigating the impact of the epidemic. We report the average values ±1 standard deviation calculated over 10,000 repetitions of each simulation, where the parameter values are sampled using a Latin Hypercube technique centred at the nominal parameter values reported in Supplementary Table 4.

## Discussion

Following the initial COVID-19 outbreak in Northern Italy, the Italian government, as many other governments around the world, adopted increasingly stricter lockdown measures at the national level to mitigate the epidemic. Despite their success, their high economic costs have stirred a hot national debate on whether such measures were necessary in the first place and on how to relax them while avoiding future epidemic waves. Several attempts have been made in the literature at addressing these pressing open issues by means of aggregate models (originated from the classical SIR model) to describe the effects of different intervention strategies at the national level^6,7^. A network model has also been recently proposed to describe the spatial dynamics of the spread of the COVID-19 epidemic among the 107 Italian provinces^14^. Other works in the literature have explored the effects of intermittent measures, either periodic or as a function of some observable quantities, as a viable alternative to long, continuous periods of national lockdown. However, the effects of these strategies have only been investigated on theoretical aggregate models at the national level^7,27^.

An important missing aspect that we considered in our study is the effect of regional heterogeneity on the efficacy of the measures taken so far and the possibility of adopting differentiated and localized intervention strategies thanks to the pseudo-federalist administrative structure of the Italian Republic. Our results confirm the effectiveness at the regional level of the national lockdown measures taken so far. They also convincingly reveal the presence of important regional effects due, for example, to the saturation of regional healthcare systems or to the presence of notable North–South flows in the country that followed the announcement of national measures. Also, contrary to previous work, we explicitly accounted for the strongly nonlinear nature of the model and the uncertainty present in the data by performing a sensitivity analysis on the estimated parameters that further confirmed the robustness of the proposed strategies for a wide range of parameter changes.

Our study strongly suggests for policy and decision-makers the potential benefits of differentiated (but coordinated) feedback regional interventions, which can be used independently or in combination with other measures, in order to avoid future epidemic waves or even to contain the outbreak of potential future epidemics. Despite having been focused on Italy, our methodology and modelling approach can be easily extended to other levels of granularity, e.g., countries in a continent or counties in a state, and adapted to any other nation where regional heterogeneity is important and cannot be neglected; notable examples are countries with a federal state organization such as Germany or the United States of America.

Future work needs to address further aspects as, for example, exploring how the structural properties of the inter-regional network can influence the dynamics of the epidemic or adopting more sophisticated cost functions to design more effective region-specific mitigation strategies in other contexts or for other purposes.

## Methods

### Regional and national model

**Fig. 5:**
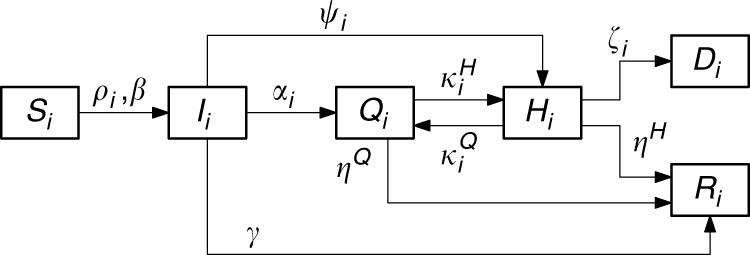
Regional compartmental model structure adopted in our study. Schematic structure of model described by Eqs. (1)–(6). Compartments describe the dynamics of susceptible (*S*_*i*_), infected (*I*_*i*_), quarantined (*Q*_*i*_), hospitalized (*H*_*i*_), recovered (*R*_*i*_) and deceased (*D*_*i*_). The number of hospitalized requiring ICU is estimated as 10% of the total. The model structure was suggested from data analysis with links between compartments being added or removed according to how the data were matched by the model (see Supplementary Notes for further details).

As a regional model of the COVID-19 epidemic spread, we use the compartmental model shown in Fig. 5, which we found from data analysis and identification trials to be the simplest model structure able to capture the real data. Specifically, we constructed the model by testing how different configurations of the links among its compartments affected the model ability to capture the available data.

The full model equations describing the dynamics of susceptible (*S*_*i*_), infected (*I*_*i*_), quarantined (*Q*_*i*_), hospitalized (*H*_*i*_), recovered (*R*_*i*_) and deceased (*D*_*i*_) are

1 $$\dot S_i = - \rho _i\beta \frac{{S_iI_i}}{{N_i}},$$

2 $$\dot I_i = \rho _i\beta \frac{{S_iI_i}}{{N_i}} - \alpha _iI_i - \psi _iI_i - \gamma I_i,$$

3 $$\dot Q_i = \alpha _iI_i - \kappa _i^HQ_i - \eta _i^QQ_i + \kappa _i^QH_i$$

4 $$\dot H_i = \kappa _i^HQ_i + \psi _iI_i - \eta _i^HH_i - \zeta _i\,H_i - \kappa _i^QH_i$$

5 $$\dot D_i = \zeta _i\,H_i,$$

6 $$\dot R_i = \gamma I_i + \eta _i^QQ_i + \eta _i^HH_i$$

where *β* and *γ* are the infection and recovery rate, respectively, which are assumed to be the same for all regions as COVID-19 is transmitted from person to person and there is no parasite vector or evidence of environmental parameters significantly altering its infection rate, *ρ*_*i*_ ∈[0, 1] is a parameter modelling the effects of social distancing measures in the *i-*th region, *α*_*i*_ is the rate of infected that are detected and quarantined, *ψ*_*i*_ is the rate of infected that needs to be hospitalized, $\eta _i^Q$ is the rate of quarantined who recover, $\eta _i^H$ is the fraction of hospitalized who recover, $\kappa _i^Q$ is the rate of hospitalized that is transferred to home isolation, $\kappa _i^H$ is the rate of quarantined who need to be hospitalized, and *ζ*_*i*_ is the mortality rate that was shown from data analysis (see Supplementary Notes) to be a function of the ratio between *H*_*i*_ and the maximum number, say $T_i^H$, of patients that can be treated in ICU at the hospitals in *i*-th region. *N*_*i*_ is the actual population in region *i*, i.e., the resident population without those removed because they had been quarantined, hospitalized, deceased or had recovered.

Extending previous approaches for modelling Dengue fever in Brazil^28^, we obtain the national network model of the COVID-19 epidemic in Italy as a network of twenty regions (see Fig. 1a) interconnected by links modelling commuter flows and major transportation routes among them.

The network model of Italy we adopt in this study is, for *i* = 1, …, 20,

7 $$\dot S_i = - \mathop {\sum}\limits_{j = 1}^M {\mathop {\sum}\limits_{k = 1}^M {\rho _j} } \beta \phi _{ij}\left( t \right)S_i\frac{{\phi _{kj}\left( t \right)I_k}}{{N_j^p}},$$

8 $$\dot I_i = \mathop {\sum}\limits_{j = 1}^M {\mathop {\sum}\limits_{k = 1}^M {\rho _j} } \beta \phi _{ij}\left( t \right)S_i\frac{{\phi _{kj}\left( t \right)I_k}}{{N_j^p}} - \alpha _iI_i - \psi _iI_i - \gamma I_i,$$

9 $$\dot Q_i = \alpha _iI_i - \kappa _i^HQ_i - \eta _i^QQ_i + \kappa _i^QH_i,$$

10 $$\dot H_i = \kappa _i^HQ_i + \psi _iI_i - \eta _i^HH_i - \kappa _i^QH_i - \zeta \left( {H_i{\mathrm{/}}T_i^H} \right)H_i,$$

11 $$\dot D_i = \zeta \left( {H_i{\mathrm{/}}T_i^H} \right)H_i,$$

12 $$\dot R_i = \gamma I_i + \eta _i^QQ_i + \eta _i^HH_i$$

13 $$N_i^p = \mathop {\sum}\limits_{k = 1}^M {\phi _{ki}} \left( t \right)\left( {S_k + I_k + R_k} \right)$$

where in addition to the parameters and states described above, we included the fluxes *ϕ*_*ij*_(*t*) between regions; $\phi _{ij}\left( t \right):{\Bbb R} \to \left[ {0,1} \right]$ denoting the ratio of people from region *i* interacting with those in region *j* at time *t*, such that $\mathop {\sum}\nolimits_j {\phi _{ij}} \left( t \right) = 1.$ Note that, as a result of the identification procedure illustrated in Supplementary Notes, in Eqs. (10) and (11) the mortality rate *ζ* is expressed as a function of the saturation of the regional health systems whose expression is given in Supplementary Notes.

### Model parameterization from real data and model validation

We divide the model parameterization into two stages. Firstly, we estimate from the available data the parameters of each of the twenty regional models; then, we use publicly available mobility data in Italy to estimate the fluxes among the regions.

As a compromise between the estimates reported in the literature on COVID-19^2,14^ in Italy (see Supplementary Table 5), we set *β* = 0.4 and *γ* = 1/14 [days^−1^] for all regions. We make the ansatz that parameters remain constant over time intervals *T*_*k*_ but do not assume the number or duration of such intervals known a priori. Therefore, we set the problem of estimating the parameters values and when they change in each region (as a likely result of national containment measures). We start estimating the parameters in each region from the first date when the number of deceased and the number of recovered is greater than or equal to 10.

Note that the official data for the COVID-19 epidemic^22^, as collected by the Dipartimento della Protezione Civile—Presidenza del Consiglio dei Ministri (the Italian Civil Protection Agency), includes for each region the daily numbers of quarantined ($\tilde Q_i$), hospitalized ($\tilde H_i$), deceased ($\tilde D_i$) and the daily number of individuals that recovered from those who were previously hospitalized or quarantined, say $\tilde R_i^O$. To fit the model to these data, we discretize and rewrite Eqs. (1)–(6) for each region (*i* = 1, …, 20) as (dropping the subscripts to the parameters for notational convenience)

14 $$\hat S_i\left( {t + 1} \right) = \hat S_i\left( t \right) - \rho \beta \frac{{\hat S_i\left( t \right)\hat I_i\left( t \right)}}{{N_i\left( 0 \right) - \tilde Q_i\left( t \right) - \tilde H_i\left( t \right) - \tilde D_i\left( t \right)}}$$

15 $$\hat I_i\left( {t + 1} \right) = \hat I_i\left( t \right) + \rho \beta \frac{{\hat S_i\left( t \right)\hat I_i\left( t \right)}}{{N_i\left( 0 \right) - \tilde Q_i\left( t \right) - \tilde H_i\left( t \right) - \tilde D_i\left( t \right)}} - \gamma \hat I_i(t) - \tau \hat I_i(t)$$

16 $$\hat C_i\left( {t + 1} \right) = \tilde C_i\left( t \right) + \tau \hat I_i\left( t \right)$$

17 $$\hat Q_i\left( {t + 1} \right) = \tilde Q_i\left( t \right) + \alpha \hat I_i\left( t \right) - \eta ^Q\tilde Q_i\left( t \right) - \kappa ^H\tilde Q_i\left( t \right) + \kappa ^Q\tilde H_i\left( t \right)$$

18 $$\hat H_i\left( {t + 1} \right) = \tilde H_i\left( t \right) + \psi \hat I_i\left( t \right) - \eta ^H\tilde H_i\left( t \right) + \kappa ^H\tilde Q_i\left( t \right) - \kappa ^Q\tilde H_i\left( t \right) - \zeta \tilde H_i\left( t \right)$$

19 $$\hat R_i^O\left( {t + 1} \right) = \tilde R_i^O\left( t \right) + \eta ^Q\tilde Q_i\left( t \right) + \eta ^H\tilde H_i\left( t \right)$$

20 $$\hat D_i\left( {t + 1} \right) = \tilde D_i\left( t \right) + \zeta \tilde H_i\left( t \right)$$

where measured quantities are denoted by a tilde and estimated state variables by a hat and *τ*: = *α* + *ψ*. Here, $C_i = Q_i + H_i + D_i + R_i^O$ represents the total number of cases detected in region *i* as daily announced by the Protezione Civile.

We notice that, exploiting the available data, the predictor can be split into two parts so that two different algorithms can then be used to estimate the parameters of each part. An ad hoc identification algorithm estimates the parameters of Eqs. (14)–(16) and automatically detects the breakpoints where notable parameter changes occur, while an ordinary least squares method is then used to identify the parameters of Eqs. (17)–(20), as described in detail in Supplementary Notes. Note that, as the actual number of infected is not known^9,29^, we include the number of infected at the beginning of each time window as a parameter to be estimated by the algorithm used for the nonlinear part.

The results of the identification process also show the presence of a statistically significant correlation (*p*-value equal to 0.071) between the value of the mortality rate, parameter *ζ*_*i*_ in model (1)–(6), and the saturation of the regional health system represented by the ratio between the number of hospitalized in that region (*H*_*i*_) and the total number of available hospital beds in intensive care ($T_i^H$) (See Supplementary Notes and Supplementary Fig. 14 for further details and function estimation).

Validation is carried out by using the parameterized model to capture the available data for each window showing a mean squared error less than 10% over the entire dataset. The parameters identified in each window can also be used to provide model predictions of future trends of the epidemic disease as discussed in Supplementary Notes.

### Cost estimation

We estimate the cost of each regional lockdown as the sum of the costs for social care and the loss of added value. The costs for social care in each region were computed as the costs for layoff support (“cassa integrazione in deroga”), estimated by multiplying the number of requests^23^ by 65% of the average regional monthly income^24^, together with the non-repayable-loan of 600 € given to self-employed workers by the Italian Government during the national lockdown^25^. The loss of added value per day was taken from the values estimated by SVIMEZ (the Italian Association for the development of Industry in the South) in Table 3 of their online report^26^. We then compute the daily costs of the lockdown and use it to estimate the total costs of each of the simulated scenarios.

### Data fitting and sensitivity analysis

All computational analyses and the fitting of data were performed using MATLAB and its optimization toolbox. To account for the inherent uncertainty associated to the COVID-19 epidemic, and hence to provide a better validation of the proposed intermittent strategies, each result reported in the manuscript is the output of 10,000 numerical simulations, where we varied the values of the model parameters using the Latin Hypercube sampling method^30^. Specifically, the regional parameters $\alpha _i,\psi _i,\kappa _i^Q,\kappa _i^H,\eta _i^Q,\eta _i^H$ together with the estimated initial conditions at May 3, 2020 $I_{f,i}$ were varied considering a maximum variation of ±20% from their nominal values (indicated in Supplementary Table 4). Our results show that the strategies we propose are robust to large parameter variations confirming, as is typical in control theory, their viability to control and mitigate the disease. Note that the model describing the epidemic spread is highly nonlinear and therefore potentially sensitive to parameter perturbations. In particular for some regions the nominal value of the basic reproduction number *R*_0_ is such that a parametric variation of 20% explores parameter sets where it becomes greater than 1, leading to dynamics that changes significantly across different simulations.

### Implementation and design of national and regional feedback intervention strategies

We model the implementation of regional social distancing strategies by capturing their effects as a variation of the social distancing parameters, *ρ*_*i*_ in (7)–(8), in each region. Specifically, we assume each region adopts the following feedback control rule with hysteresis:

- *ρ*_*i*_ is set and kept equal to $\underline \rho _i$ as long as the saturation of the regional health system, computed as the ratio between the number of the hospitalized requiring care in ICU (estimated as 0.1*H*_*i*_) over the number of available ICU beds in the region, is above 20%, i.e., $\rho _i = \underline \rho _i,{\mathrm{if}}\,\frac{{0.1H_i}}{{T_i^H}} \ge 0.20$
- *ρ*_*i*_ is set and kept equal to $\bar \rho _{\mathrm{i}}$ as long as the saturation of the regional health system is below 10%, i.e., $\rho _i = \bar \rho _i,{\mathrm{if}}\,\frac{{0.1H_i}}{{T_i^H}} \le 0.10$

In our simulations, $\underline \rho _i$ is set equal to the minimum estimated value in that region during the national lockdown (see Supplementary Table 4) and $\bar \rho _{\mathrm{i}}$ increased as a worst case to ${\mathrm{min}}(1,3\underline \rho _i)$ so as to simulate the effect of relaxing the lockdown measures in each region. (The case where $\bar \rho _{\mathrm{i}}$ is set to a lower value equal to 1.5$\underline \rho _i$ is shown for the sake of comparison in Supplementary Figs. 5 and 6.)

Also, when a region is in lockdown, we assume all fluxes in and out of that region are reduced by 70% of their original values to better simulate the actual reduction in people’s movement observed during the lockdown in Italy (for further details see Supplementary Information). Such a reduction level was estimated qualitatively by considering the publicly available mobility data from Google (Google mobility data (https://www.google.com/covid19/mobility/)).

National lockdown measures are modelled by setting all *ρ*_*i*_ simultaneously to $\underline \rho _i$ in all regions and reducing all fluxes by 70% while national reopening of all regions by setting all *ρ*_*i*_ simultaneously to $\bar \rho _{\mathrm{i}}$ and restoring inter-regional flows to their prelockdown level.

To model the increase in the COVID-19 testing capacity of each region the parameter *α*_*i*_ in region *i* is multiplied by a factor 2.5, which corresponds to the average increase in the number of tests carried out nationally since the COVID-19 outbreak first started.

### Numerical simulations

All simulations were carried out in MATLAB with a discretization step of 1 day to match the available data sampling interval. Initial conditions for regional compartments were set as follows. Quarantined, Hospitalized, Deceased and Recovered are initially set to the datapoints available for May 3, 2020. The number of infected is set to the value *I*_*f*_ estimated by our procedure for that date; Susceptibles are initialized to the resident population from which the other compartments are removed.

Further details are given in Supplementary Notes.

### Reporting summary

Further information on research design is available in the Nature Research Reporting Summary linked to this article.

## Supplementary information

Supplementary Information

Reporting Summary

## Data Availability

The authors declare that the data supporting the findings of this study are available within the paper and its Supplementary Information files or from the corresponding author on reasonable request. The source data for all figures in the main text and Supplementary Information are provided as a Source Data file at https://github.com/diBernardoGroup/Network-model-of-the-COVID-19.

## Code availability

The code to run all simulations and the model is available at https://github.com/diBernardoGroup/Network-model-of-the-COVID-19.
