# Supplementary material for: A network model of Italy shows that intermittent regional strategies can alleviate the COVID-19 epidemic
Source: Nat Commun. 2020 Oct 9;11:5106. doi: 10.1038/s41467-020-18827-5 (PMC7547104; doi:10.1038/s41467-020-18827-5)
Supplement: Supplementary file 1 — Supplementary Information [file 41467_2020_18827_MOESM1_ESM.pdf]

SUPPLEMENTARY INFORMATION

A network model of Italy shows that intermittent regional strategies can alleviate the COVID-19 epidemic

Fabio Della Rossa[*,a], Davide Salzano[*,b], Anna Di Meglio[*,b], Francesco De Lellis[*,b], Marco Coraggio[b], Carmela Calabrese[b], Agostino Guarino[b], Ricardo Cardona-Rivera[b], Pietro De Lellis[b], Davide Liuzza[c], Francesco Lo Iudice[b], Giovanni Russo[d], Mario di Bernardo[b,#]

[a]*Department of Electronic, Information and Biomedical Engineering, Politecnico di Milano, Italy*

[b]*Department of Electrical Engineering and Information Technology, University of Naples Federico II, Italy*

[c]*ENEA, Fusion and Nuclear Safety Department, Frascati (Rome), Italy*

[d]*Department of Information and Electrical Engineering and Applied Mathematics, University of Salerno, Italy*

* *These authors contributed equally*

# Correspondence: mario.di.bernardo@unina.it

Supplementary Notes **Page**

Supplementary Note 1: Data analysis 3

Supplementary Note 2: Identification procedure 5

Supplementary Note 3: Regional feedback intervention strategies 12

Supplementary Note 4: Further details on the numerical code 13

Supplementary References 14

Supplementary Figures

Supplementary Figure 1: Only one region relaxes its lockdown 16

Supplementary Figure 2: All regions relax their lockdown measures 17

Supplementary Figure 3: National lockdown 18

Supplementary Figure 4: Regional dynamics when intermittent national measures are enforced 19

Supplementary Figure 5: Intermittent regional measures 20

Supplementary Figure 6: Intermittent national measures 21

Supplementary Figure 7: Intermittent regional measures with increased COVID-19 testing capacity and $\bar{\rho}_i = 1.5\underline{\rho_i}$ 22

Supplementary Figure 8: Networks resulting from the estimation of flows 23

Supplementary Figure 9: Identification of the aggregate national model 24

Supplementary Figure 10: Example of data matching and prediction ability 24

Supplementary Figure 11: Identification of the regional models - Steps 1,2 25

Supplementary Figure 12: Identification of the regional models - Step 3 26

Supplementary Figure 13: Distribution of the social distancing parameter over time 27

Supplementary Figure 14: Fitting of ζ_i as a function of $\overline{H}_i$ 27

Supplementary Tables

Supplementary Table 1: Comparison of each of the simulated scenarios over 1 year 28

Supplementary Table 2: Constraints for the regional models' parameterization 29

Supplementary Table 3: Parameters of the aggregate national model 30

Supplementary Table 4: Model parameters 31

Supplementary Table 5: Comparison with the parameters estimated in other papers in the literature for the national aggregate models 32

Supplementary Note 1: Data analysis

Data on the evolution of the epidemic in Italy

Data on the evolution of the epidemic in Italy and in each of the 20 regions have been obtained from the public database[7] of the Italian Civil Protection Agency (Protezione Civile). The database provides daily updates for each region on the overall number of detected cases, hospitalized, quarantined, and recovered. The data were pre-processed by filtering them with a moving average filter of 3 days or 7 days to reduce noisiness. Specifically, a moving average filter of 3 days was used to preprocess data for all regions but for Valle d'Aosta, Umbria and Basilicata. For these three regions, which presented higher levels of noise, we used a longer average filter of 7 days. These lengths were chosen heuristically so as to best capture the parameters and their variation among different time windows.

Interregional Fluxes estimation

We considered two types of inter-regional fluxes associated to

(i) daily commuters traveling between neighboring regions;

(ii) long distance travels covered by high-speed trains, planes, and large ferries.

To estimate commuters' fluxes, as in previous work[6], we used the latest official country-wide assessment of Italian mobility conducted by the Italian Institute of Statistic (ISTAT) in 2011. Specifically, we use the origin-destination matrix describing, at the municipality level, the number of people who declared themselves daily commuters for work or study purposes (https://www.istat.it/it/archivio/139381). By aggregating data on a regional basis, we obtained interregional commuters' flows among regions.

For longer distance routes covered by high-speed trains, planes and large ferries we proceeded as follows.

a. For railway connections, the fraction ϕ_{ij}^r of the population of region i traveling to region j is obtained as

$$\phi_{ij}^r = \frac{1}{p_i} \frac{\phi^r}{n^r} n_{ij}^r,$$

where

- ϕ^r is total number of daily high-speed customers of the main Italian carrier, Trenitalia;
- n^r is the total number of high-speed trains per day;
- n_{ij}^r is the number of high-speed trains connecting region i to region j;
- p_i is the total resident population in region i.

b. For flight connections, for all the Italian operating airports (currently there are 39 according to www.enac.gov.it) and for each pair of connected airports, say (l, k), we used the number of *weekly* direct flights f_{lk} in normal operating conditions (i.e., without any restriction due to the emergency). Then, for each pair (l, k) we computed the average capacity $\langle c_{lk} \rangle$ of the regional fleet of the main carrier serving the route. Grouping the airports on a regional basis we computed the average daily flow due to air connections ϕ_{ij}^a from region i to j as:

$$\phi_{ij}^a = \frac{\sum_{l=1}^{N_i} \sum_{k=1}^{N_j} f_{lk} \langle c_{lk} \rangle}{7 p_i},$$

where N_i and p_i are the number of airports and the population of region i.

c. For ferry connections, we considered the five regions that act as hubs for long range national maritime travel, that is, the two main insular regions, Sardinia and Sicily, together with three mainland regions, Campania, Lazio, and Liguria. The maritime flows are then obtained as

$$\phi_{ij}^m = \frac{\phi_i}{p_i \, n_{ij}^m},$$

where ϕ_i and p_i are the average number of maritime passengers and the population of region i respectively, while n_{ij}^m is the total number of maritime connections between regions i and j. Note that as it is reasonable to assume that $n_i \phi_{ij} \approx n_j \phi_{ji}$, and as all relevant maritime routes are either from or to the main islands, in practice, it suffices to compute ϕ_{ij}^m only for region i being Sicily and Sardinia.

Note that, as also done in other network models in the literature[6,27], we chose not to consider long term migrations as they generally occur on a longer time-scale than that we are interested in to characterize the COVID19 epidemic in Italy. Looking at available data on fluxes among regions over the timespan of interest we noted that the fraction of people permanently moving from a region to another is negligible compared to the total resident population in the region. Hence, our model, as also done in other papers[6,27], takes into account commuters fluxes from

one region to the other; the infected of a region contributing to increasing the likelihood of generating new infected in the region they move to.

Supplementary Note 2: Identification procedure

As explained in the main text, we assume that, for each region, the parameters of the model remain constant over n time windows, but neither their number n nor their durations $\delta_1, \dots, \delta_n$ are assumed to be known *a priori*. Therefore, the identification procedure detects at the same time the breakpoints $t_1, \dots, t_{n-1}$ when notable parameters' changes are detected and, within each time-window, estimates their values as those that best capture the trend of the available data.

The model used to carry out the identification of regional or national parameters is the discretized version of the model of the epidemic spread in each area of interest given by model (14)-(20) in the main text.

As also noted in other previous work[1-4], identification of SIR and SIR-modified models is highly non-convex and hence the optimization landscape is scattered with local minima that must be avoided as not being admissible. To mitigate this problem, we identified from the literature admissible intervals for the parameter values (see Supplementary Table 5) in order to reduce the feasible search space for the optimization algorithm and provide it with reasonable initial guesses. Note that, β and ρ always appear as a product in the model. Therefore, only their product can be identified. Hence, we fixed β to an intermediate value of 0.4 from those reported in the literature[3,6], where the estimates for β range from 0.301 [3], 0.373 [15], and 0.315 [14] to a much higher value of 1.12 [17] (when β models the transmission rate for documented infections and therefore can become larger than 1 capturing the fact that the new infected can get the disease from undetected infected individuals[17]). We found that setting β =0.4 in our model roughly scales the parameter ρ between 0 and 1, making apparent the effect of the social distancing rules imposed during the lockdown. Moreover, since γ represents the flux of infected people that recover without having any symptoms, thus connecting two compartments that cannot be measured, we chose to fix this rate at 1/14 days^{-1} as a conservative approximation of the values reported in recent work[6] where the longest estimate of the infectious period for an asymptomatic infected (counting from as soon as the contagion occurs) is 12.93 days. Note that in other works[3] an even higher choice of 29.41 days is used. Altogether, the unknown parameters left to be estimated are $[I(0), \rho, \tau, \alpha, \eta^Q, \eta^H, \kappa^H, \kappa^Q, \zeta]$ both at the national and regional level.

As mentioned in the main text, the identification procedure is carried out in two stages by considering equations (14)-(16) of the main text to estimate $\{\tau, I(0), \rho\}$ and the window

breakpoints, and equations (17)-(20) of the main text to estimate the remaining parameters. The identification described next is repeated for each of the 20 regions and, for the sake of completeness, to parameterize a national aggregate model.

Step 1: Online Identification of the estimation breakpoints and the parameters $\tau, I(0), \rho$ in each time window

We start by identifying the parameters' vector $\boldsymbol{\theta} := [I(0), \rho, \tau]$ exploiting equations (14)-(16) of the main text and the time series of the number of cases $\tilde{C}$ collected for T_{tot} consecutive days starting from the day when 10 deceased and 10 recovered were first reported in the area of interest. In particular, an *ad hoc* optimization algorithm (described below and implemented in MATLAB) is used to find breakpoints t_j and the values of the parameters' vector $\widehat{\boldsymbol{\theta}}$ that minimize the cumulative squared prediction error in each window, defined as

$$SSE\left(\widehat{\boldsymbol{\theta}}, t_j, t_{j+1}\right) = \sum_{t=t_j}^{t_{j+1}} \left\| \tilde{C}(t, \boldsymbol{\theta}) - \hat{C}\left(t, \widehat{\boldsymbol{\theta}}\right) \right\|^2$$

with $j = 0, 1, 2, \dots, n-1$.

We use the following recursive procedure:

1. Set the initial time t_0 as the first day in which the first 10 deaths and 10 recovered were reported in the area (region or nation).
2. Assume the initial guess for the width of the window to be $T = \lceil (2p+1)/d \rceil$, where p is the number of parameters to be identified and d the number of measured variables.
3. Estimate the parameters over the entire window to obtain
$$\widehat{\boldsymbol{\theta}} = \underset{\widehat{\boldsymbol{\theta}}}{\operatorname{argmin}}\, SSE\big(\widehat{\boldsymbol{\theta}}, t_0, t_0 + T\big)$$
4. Divide the window into two intervals and estimate the parameters over each subsample obtaining the two estimates
$$\widehat{\boldsymbol{\theta}}_a = \underset{\widehat{\boldsymbol{\theta}}_a}{\operatorname{argmin}}\, SSE\big(\widehat{\boldsymbol{\theta}}_a, t_0, t_0 + \lceil T/2 \rceil\big), \text{ and } \widehat{\boldsymbol{\theta}}_b = \underset{\widehat{\boldsymbol{\theta}}_b}{\operatorname{argmin}}\, SSE\big(\widehat{\boldsymbol{\theta}}_b, t_0 + \lceil T/2 \rceil, t_0 + T\big).$$
5. Perform Chow statistical test
$$F = \frac{(T-2p)(\sigma - (\sigma_a + \sigma_b))}{p(\sigma_a - \sigma_b)} \sim \mathcal{F}_{\{p,T-2p\}}$$
where
$$\sigma = SSE\big(\widehat{\boldsymbol{\theta}}, t_0, t_0 + T\big)$$
$$\sigma_a = SSE\big(\widehat{\boldsymbol{\theta}}_a, t_0, t_0 + \lceil T/2 \rceil\big)$$
$$\sigma_b = SSE\big(\widehat{\boldsymbol{\theta}}_b, t_0 + \lceil T/2 \rceil, t_0 + T\big)$$
with null hypothesis $H_0\colon \big\{\widehat{\boldsymbol{\theta}}_1 = \widehat{\boldsymbol{\theta}}_2\big\}$ and critical p-value $p^* = 10^{-5}$.
6. Then,
    a. if $\mathcal{F}_{\{p,T-2p\}}(F) > p^*$, the null hypothesis cannot be rejected, and the parameters are considered constant in the time-window T. Then, the length of the current window is increased by setting $T = T + 1$, and steps 3, 4 and 5 are repeated;
    b. if $\mathcal{F}_{\{p,T-2p\}}(F) \leq p^*$, the null hypothesis is rejected, and then the next breakpoint t_1 is selected as
$$t_1 = \operatorname{argmax} \mathcal{F}_{\{p,T-2p\}}(F)$$
   and the parameter set $\widehat{\boldsymbol{\theta}}$ that minimizes $SSE\big(\widehat{\boldsymbol{\theta}}, t_0, t_1\big)$ is selected as the set that best fits the data over the window (t_0, t_1) whose duration is therefore $\delta_1 := t_1 - t_0$.
7. If $t_1 = T_{\text{tot}}$ the algorithm is stopped, otherwise starting from t_1 steps 2-7 are repeated to find the next breakpoint and the new set of parameters best estimating the data in the next window until the end of the available datapoints.

Step 2: Offline refinement of the identification process

At the end of the process we will have the set of breakpoints t_j and the parameters set in each of the windows (t_j, t_{j+1}) best fitting the data. As the number of windows can be large given the variability in the available data, we refine the estimation results as follows to estimate the minimal number of windows able to capture qualitatively the trend of the real data.

In particular, once the window breakpoints are obtained at the end of step 1, any two consecutive windows of duration say δ_j, δ_{j+1} are merged into one larger window of size $\delta_j + \delta_{j+1}$ if one of the two following conditions is verified:

a) The window size of the first window is less than 5 days.
b) The relative variation of the sum $\tau + \rho$ as estimated in each of the two windows is less than 5%, i.e.

$$\frac{(\tau + \rho)^{j+1} - (\tau + \rho)^{j}}{(\tau + \rho)^{j}} \leq 0.05$$

where j denotes the window to which the parameter estimates refers to.

If two windows are merged, then the parameters are estimated again on the entire merged window and the procedure is iterated once more in case condition b) is still verified.

As a final refinement step, we heuristically explore the effect on the fitting of perturbing the breakpoints within ±5 days from their estimated value. As a representative example, the results of the fitting procedure for the national aggregate model are shown in Supplementary Table 3 and depicted in Supplementary Figure 9. The same procedure is repeated to parametrize each of the 20 regional models.

As the key use of the identified model is to validate the intermittent mitigation strategies we propose in the paper, it is crucial to check whether the proposed method overfits the data, thus worsening the model prediction ability. To provide a representative validation of our estimation approach, we report in Supplementary Figure 10 the time evolution of the total number of detected cases at the national level predicted by the model in each time window. It is possible to see that using just 30% of the datapoints from all the available data (shown as red circles), the model predictions (solid blue lines) fit well the rest of the data in each time window both before and after the windows are merged as a result of step 2 with a maximum

prediction error of 10,000 units. A further numerical validation of the time window estimation algorithm is provided later in this Note.

Step 3: Identifying the parameters $\eta^H, \eta^Q, \psi, \alpha, \kappa^H, \kappa^Q, \zeta$

For each of the time windows identified in Step 2, using the time series $\hat{I}(t)$ estimated from the equations parametrized in Step 1, and considering that equations (17)-(20) of the predictor reported in the main text are linear with respect to the parameters, we use an ordinary constrained least squares method, with constraints given in Supplementary Table 2 to compute the remaining parameters.

The comparison between the model predictions and the available data is depicted in Supplementary Figure 11 and Supplementary Figure 12. Values of all estimated parameters at the end of the process are given in Supplementary Table 4 for each region where in the last column the regional net reproduction numbers are computed as $R_{0,i} = \rho_i \beta / (\alpha_i + \psi_i + \gamma)$ in each time window. Already in the earliest windows it is possible to see the effects of the first measures taken by the government that date back to February 23rd and March 4th. Indeed, estimates of $R_{0,i}$ in the first windows are lower than the value between 3 and 4 estimated in the literature[2]. Taking Lombardy as a representative example and carrying out our identification procedure on the first 7 days (from February 24th to March 1st), yields an estimate of $R_0 = 3.37$, for that region, confirming that the social distancing measures adopted by the government started taking effect around the beginning of March.

Note that we enforce continuity of the trajectory between different time windows by imposing soft constraints in the optimization problems (see Supplementary Table 2) so that the result of the simulation in the previous time window constrains the dynamics in the next one. The apparent discontinuity between time-windows that can be observed in the parameter values given in Supplementary Table 4 is typical of all predictor-corrector algorithms where after a certain number of prediction steps the actual data points are used to "correct" the final predictions and restart over the next period. This is standard in systems and control theory (e.g. Kalman predictor and *n*-step ahead predictors).

Supplementary Figure 13 shows the distribution of the regional social distancing parameters over time depicting the effects of the national lockdown at the regional level.

ζ_i as a function of the occupancy of ICU beds in each region

Observing the data and the identified parameters in Supplementary Table 4, we noticed a significant correlation between the mortality rate in each time window and the congestion of the ICU system in that region.

Specifically, we found that

$$\zeta_i = f(\bar{H}_i) = \zeta_0^{IC} + \zeta_1^{IC}\bar{H}_i$$

where ζ_0^{IC} and ζ_1^{IC} are coefficients to be estimated, while $\bar{H}_i$ is the estimated average congestion of the hospitals in each time-window defined as the average ratio between the number of hospitalized subjects and the number of available beds in ICU in that region, say T_i^H (obtained by linearly interpolating the number of ICU beds at the beginning of the year, reported on the web-page of the Italian Ministry of Health[8], and those reported by the Italian Government at the end of the national lockdown[9]). From a qualitative viewpoint, this assumption can be explained by the fact that the quality of the care hospitals can provide degrades as the health system becomes more and more under stress.

Each point in Supplementary Figure 14 is the value of ζ_i estimated for a given region in each time window plotted against the number of hospitalized in the corresponding region averaged over the time window. As illustrated in Supplementary Figure 14, a least square linear fitting yields $\zeta_0^{IC} = 0.016, \zeta_1^{IC} = 0.00068$. The function is then saturated at both ends, i.e. at 0 and at the value 0.023 for $\bar{H}_i \geq 10$.

Remark on the ad-hoc identification method in the context of the existing literature

As in other available methods in the literature (quasi-linearization[20], Finite Differences, Integration of Data[21] and Smooth-the-Data methods[22-25]) that cannot rely on analytical solutions of the model equations, we carried out the identification by using some approximating solution of the model predictions. Here, we integrated numerically the model using a Runge-Kutta algorithm and took the solution as a piecewise differentiable approximation of its true solutions (rather than other approximating solutions adopted in existing methods that we found unsuitable in our case). We then incorporated techniques

from the literature, as the Chow test[25,26], to find the breakpoints, that is, the points in time where the parameters change significantly.
Overall, our method is therefore in line with the others available in the literature but is fine tuned and adapted to the specific case of interest also to render its use possible online, i.e. as new datapoints become available.

Validation of the ad-hoc window estimation method on a synthetic dataset
As a further validation of its effectiveness, we tested our parameter ad-hoc window estimation algorithm over 100 synthetic datasets containing 40 datapoints each.
Each dataset is obtained by simulating a replica of model (14)-(16) given by

$$\dot{S} = -\rho\beta\frac{S\,I}{N},$$

$$\dot{I} = \rho\beta\frac{S\,I}{N} - \tau I - \gamma I,$$

$$\dot{C} = \tau I$$

$$\dot{N} = \tau I + \gamma I$$

with parameter values set equal to $\rho = 0.4, \tau = 0.1$, $\beta = 0.4$, $\gamma = 1/14, N(0) = 10^4, I(0) = 100, S(0) = N(0) - I(0), C(0) = 0$, artificially varying parameter ρ twice (after 10 time units and 20 time units respectively) reducing its value by 10% each time and adding to the output $C(t)$ of the model a uniform noise with standard deviation comparable to the values of the daily increment in the number of cases, i.e. considering as the model output the variable

$$\tilde{C}(t) := C(t-1) + u(t)\big(C(t) - C(t-1)\big)$$

with $u(t) \sim \mathcal{U}(0,2)$. Each dataset therefore contains two artificial parameter changes and should therefore be partitioned into 3 time-windows by the algorithm.
To check the rate of success of the method, we then ran the identification procedure on all the synthetic datasets that were generated, estimating the number of windows. Specifically, for each instance of the generated data, we collected the number of time windows detected by the algorithm after every step of the identification procedure described above. We found that, before the offline refinement step of the algorithm (Step 1 of the identification procedure), the algorithm detects the correct number of windows in 63% of the cases, overestimating it in 30% of the cases. However, after the offline refinement stage (Step 2 of the identification procedure), the algorithm overestimates the number of windows (i.e., over-parameterized the model) only in 3% of the cases confirming that the model is not being overfitted by the procedure. This is also confirmed, as stated above, by its good predictive ability as shown in Supplementary Figure 10.

Supplementary Note 3. Regional feedback intervention strategies

We considered three different types of intervention strategies at the regional level that can be used individually or in combination.

1) *Feedback Social distancing rule.* Each region modulates its lockdown measures so as to switch them on or off according to the relative saturation level of its health system. Namely, the social distancing parameter is modulated by the following hysteretic control rule:

$$\rho_i = \begin{cases} \underline{\rho_i}, & \text{if } \dfrac{0.1H_i}{T_i^H} \geq 0.2 \\ \bar{\rho}_i, & \text{if } \dfrac{0.1H_i}{T_i^H} \leq 0.1 \end{cases}$$

where $\underline{\rho_i}$ is set equal to the minimum estimated value in that region during the national lockdown (corresponding to the value given in the last window for each region reported in Supplementary Table 4) and, as a worst-case scenario, $\bar{\rho}_i$ is set to three times $\underline{\rho_i}$ (or unity if $3\underline{\rho_i}$ >1) to simulate a relaxation of the containment measures in that region.

2) *Feedback flux control.* Here the fluxes in or out of a region are modulated according to the following hysteretic rules

$$\phi_{ij} = \begin{cases} \underline{\phi_{ij}}, & \text{if } \frac{0.1H_i}{T_i^H} \geq 0.2 \\ \bar{\phi}_{ij}, & \text{if } \frac{0.1H_i}{T_i^H} \leq 0.1 \end{cases}, \forall j \neq i$$

and

$$\phi_{ji} = \begin{cases} \underline{\phi_{ji}}, & \text{if } \frac{0.1H_i}{T_i^H} \geq 0.2 \\ \bar{\phi}_{ji}, & \text{if } \frac{0.1H_i}{T_i^H} \leq 0.1 \end{cases}, \forall j \neq i$$

where we denote by $\underline{\phi_{ij}}$ and $\bar{\phi}_{ij}$ respectively the quarantine (low) and post quarantine (high) values of the flux from region i to region j. In particular during a lockdown we set $\underline{\phi_{ij}} = 0.3\bar{\phi}_{ij}$ while $\bar{\phi}_{ij}$ correspond to the fluxes from region i to region j estimated as described in Supplementary Notes 1.

Note that all people resident in region i not commuting to any other region are assumed to stay and move in region i itself by setting $\phi_{ii} = 1 - \sum_{j \neq i} \phi_{ij}$.

Supplementary Note 4. Further details on the numerical code

All the numerical analyses presented in the paper were performed with MATLAB. The code is available at https://github.com/diBernardoGroup/Network-model-of-the-COVID-19 and was designed to

1) Load the estimated parameter values and the inter-regional fluxes and to iterate the discretized model dynamics of each region considering the presence of interregional fluxes (MATLAB script "siqhrd_network_main.m"). The script also computes the regional and national reproduction numbers.

2) Implement the differentiated regional intervention strategies described in Supplementary Notes 3.

3) Carry out parameter sensitivity analysis by using the Latin Hypercube sampling to explore the parameter region surrounding the estimated nominal parameter values. A variation of up to 20% of all parameters was considered in the simulations reported in the paper (MATLAB scripts "siqrhd_network_main_montecarlo.m" and "hypercube_gen.m")

Further details can be found in the accompanying README.TXT file included with the code in the software repository link above.

Supplementary References

[1] Mummert, A., & Otunuga, O. M. "Parameter Identification for a Stochastic SEIRS Epidemic Model: Case Study Influenza". *Journal of Mathematical Biology*, **79**(2), p.705–729, 2019.

[2] Liu, Y., Gayle, A. A., Wilder-Smith, A., & Rocklöv, J. "The Reproductive Number of COVID-19 Is Higher Compared to SARS Coronavirus". *Journal of Travel Medicine*, **27**(2), 2020.

[3] Giordano, G., Blanchini, F., Bruno, R., Colaneri, P., Di Filippo, A., Di Matteo, A., & Colaneri, M. "Modelling the COVID-19 Epidemic and Implementation of Population-wide Interventions in Italy". *Nature Medicine*, **26**, p.1–6, 2020.

[4] Calafiore, G. C., Novara, C., & Possieri, C. A, "A Modified SIR Model for the COVID-19 Contagion in Italy". arXiv:2003.14391, (2020).

[5] Diekmann, O., Heesterbeek, J. A., & Metz, J. A., "On the Definition and the Computation of the Basic Reproduction Ratio R_0 in Models for Infectious Diseases in Heterogeneous Populations". *Journal of Mathematical Biology* **28**, p.365-382 ,1990.

[6] Gatto, M., Bertuzzo, E., Mari, L., Miccoli, S., Carraro, L., Casagrandi, R., & Rinaldo, A. "Spread and dynamics of the COVID-19 Epidemic in Italy: Effects of Emergency Containment Measures". *Proceedings of the National Academy of Sciences*, **117**(19), 2020.

[7] https://github.com/pcm-dpc/COVID-19/tree/master/dati-regioni

[8] http://www.dati.salute.gov.it/dati/dettaglioDataset.jsp?menu=dati&idPag=17

[9] https://www.trovanorme.salute.gov.it/norme/renderNormsanPdf?anno=2020&codLeg=74348&parte=1%20&serie=null

[10] Epicentro ISS, https://www.epicentro.iss.it/coronavirus/sars-cov-2-decessi-italia#2.

[11] Epidemic Calculator, https://gabgoh.github.io/COVID/index.html.

[12] Ferguson, N. M., et al., "Impact of non-pharmaceutical interventions (NPIs) to reduce COVID- 19 mortality and healthcare demand" p. 20, 2020.

[13] Peng, L., Yang, W., Zhang, D., Zhuge, C. & Hong, L., "Epidemic analysis of COVID-19 in China by dynamical modeling" Epidemiology, preprint, Feb. 2020.

[14] Casella, F., "Can the COVID-19 epidemic be controlled on the basis of daily test reports?", IEEE Control Systems Letters, 5(3), 1079-1084, 2020.

[15] Bin, M., et al., "On Fast Multi-Shot COVID-19 Interventions for Post Lock-Down Mitigation". arXiv:2003.09930, Apr. 2020.

[16] Flaxman, S., et al., "Estimating the effects of non-pharmaceutical interventions on COVID-19 in Europe", Nature, 584, 257–261, 2020.

[17] Li, R., et al., "Substantial undocumented infection facilitates the rapid dissemination of novel coronavirus (SARS-CoV2)" Science, p. eabb3221, Mar. 2020.

[18] Verity, R., et al., "Estimates of the severity of coronavirus disease 2019: a model-based analysis" The Lancet Infectious Diseases, p. S1473309920302437, Mar. 2020

[19] http://github.com/pcm-dpc/COVID-19/tree/master/dati-andamento-nazionale.
[20] Hemker, P. W., & Kok J., "A project on parameter identification in reaction kinetics." Centrum voor Wiskunde en Informatica, 1993.
[21] Bard, Y., "Nonlinear parameter estimation.", Academic Press, 1974.
[22] Scitovski, R., Ungar, Š., Jukić, D., & Crnjac, M."Moving total least squares for parameter identification in mathematical model." Operations Research Proceedings, 1995.
[23] Scitovski,R., "Analysis of a parameter identification problem." Applied mathematics and computation, **82**(1),1997.
[24] Scitovski, R. & Jukic, D."Total least-squares problem for exponential function." Inverse problems, **12**(3), 1996.
[25] Beck, N., "Time-varying parameter regression models." American Journal of Political Science, 1983.
[26] Bates, D. M., & Watts, D. G., "Nonlinear regression analysis and its applications", Wiley, 1988.
[27] Stolerman, L. M., Coombs, D., & Boatto, S. SIR-Network Model and its Application to Dengue Fever. *SIAM Journal on Applied Mathematics* **75**, p. 2581–2609, 2015.

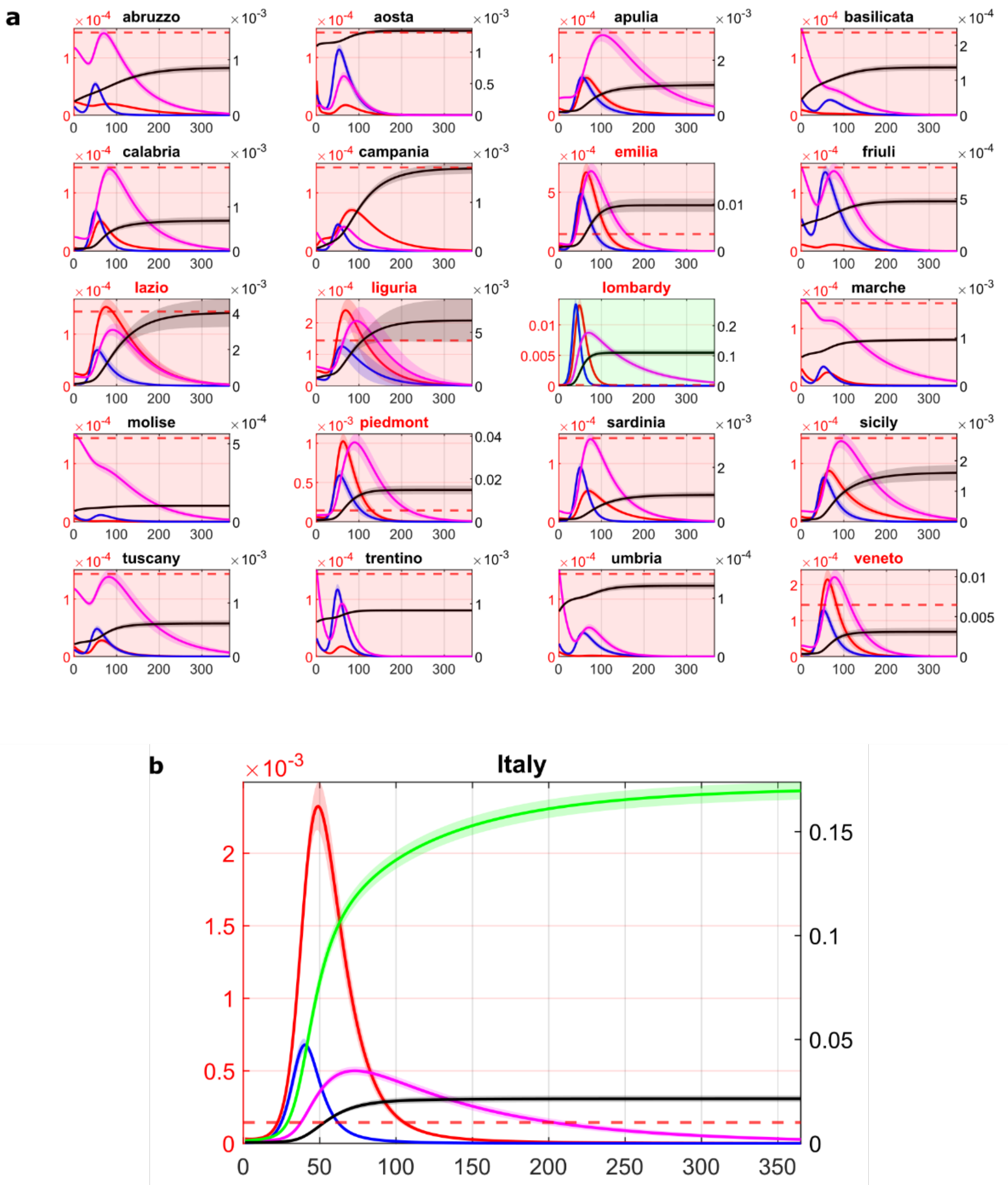

Supplementary Figure 1 [Only one region relaxes its lockdown] a. Regional and **b.** national dynamics in the case where only one region (Lombardy in Northern Italy) relaxes its containment measures at time 0 and the fluxes between regions are set to their pre-lockdown level. Panels of regions adopting a lockdown are shaded in red while those of regions relaxing social containment measures are shaded in green. Blue, magenta, red, green, and black solid lines correspond to the fraction in the population of infected, quarantined, hospitalized requiring ICU, recovered, and deceased averaged over 10,000 simulations with parameters sampled using a Latin Hypercube technique around their nominal values set as those estimated in the last time window for each region as reported in Supplementary Table 4. Shaded bands correspond to twice the standard deviation. The red dashed line identifies the total fraction of the population that can be treated in ICU (T_i^H/N_i). The regions identified with a red label are those where the total hospital capacity is saturated. All plots are shown with a double scale. The scale on the left axis (in red) applies to the hospitalized requiring ICU and the ICU beds capacity threshold (dashed red line), while the right axis (in black) applies to the infected, quarantined, recovered and deceased.

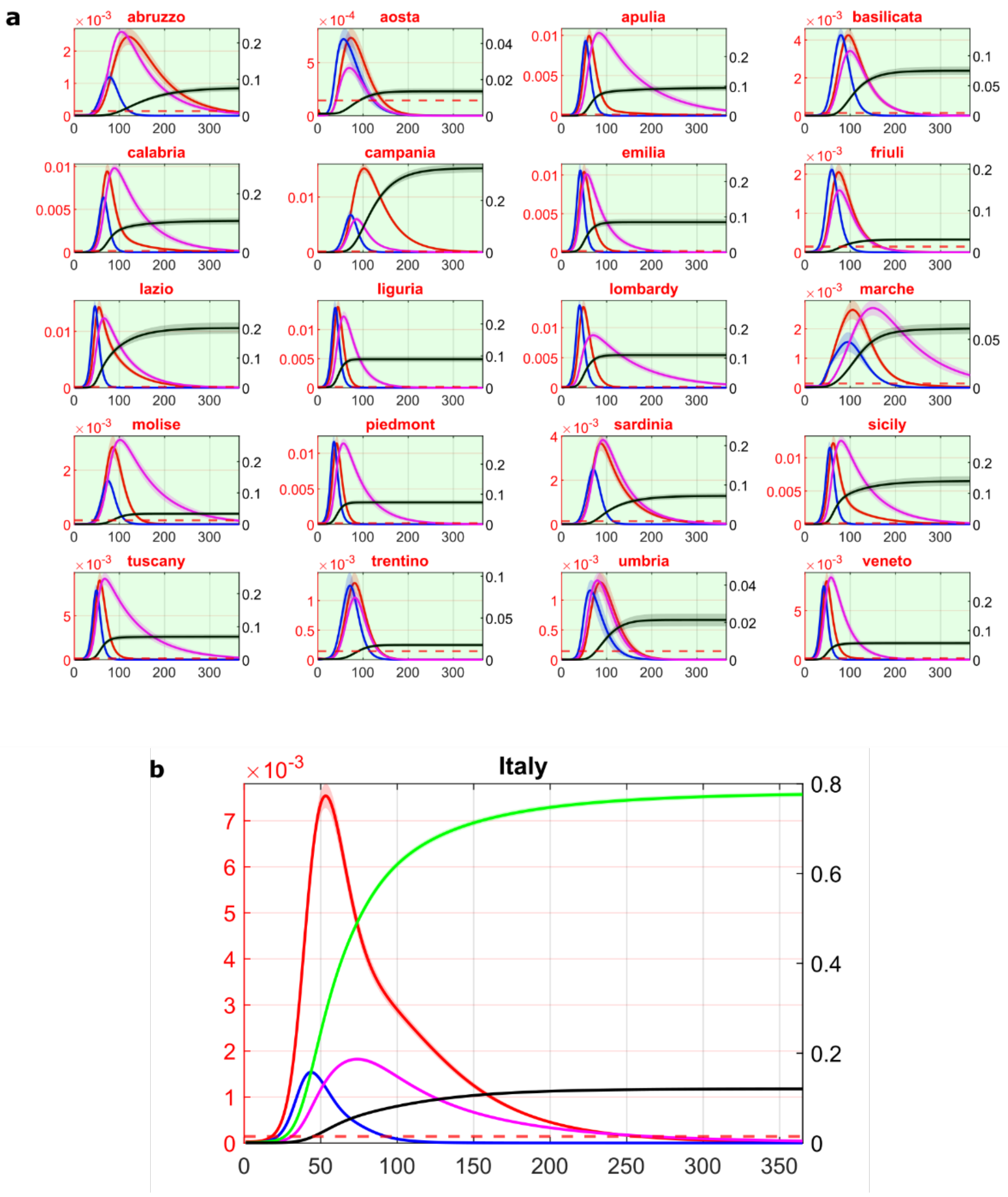

Supplementary Figure 2 [All regions relax their lockdown measures] a. Regional and **b.** national dynamics in the case where all regions relax their current restrictions restoring fluxes to their pre-lockdown level. Blue, magenta, red, green, and black solid lines correspond to the fraction in the population of infected, quarantined, hospitalized requiring ICU, recovered, and deceased in the population averaged over 10,000 simulations with parameters sampled using a Latin Hypercube technique around their nominal values set as those estimated in the last time window for each region as reported in Supplementary Table 4. Shaded bands correspond to twice the standard deviation. The red dashed line identifies the fraction of the population that can be treated in ICU (T_i^H/N_i). The regions identified with a red label are those where the total hospital capacity is saturated. All plots are shown with a double scale. The scale on the left axis (in red) applies to the hospitalized requiring ICU and the ICU beds capacity threshold, while the right axis (in black) applies to the infected, quarantined, recovered and deceased.

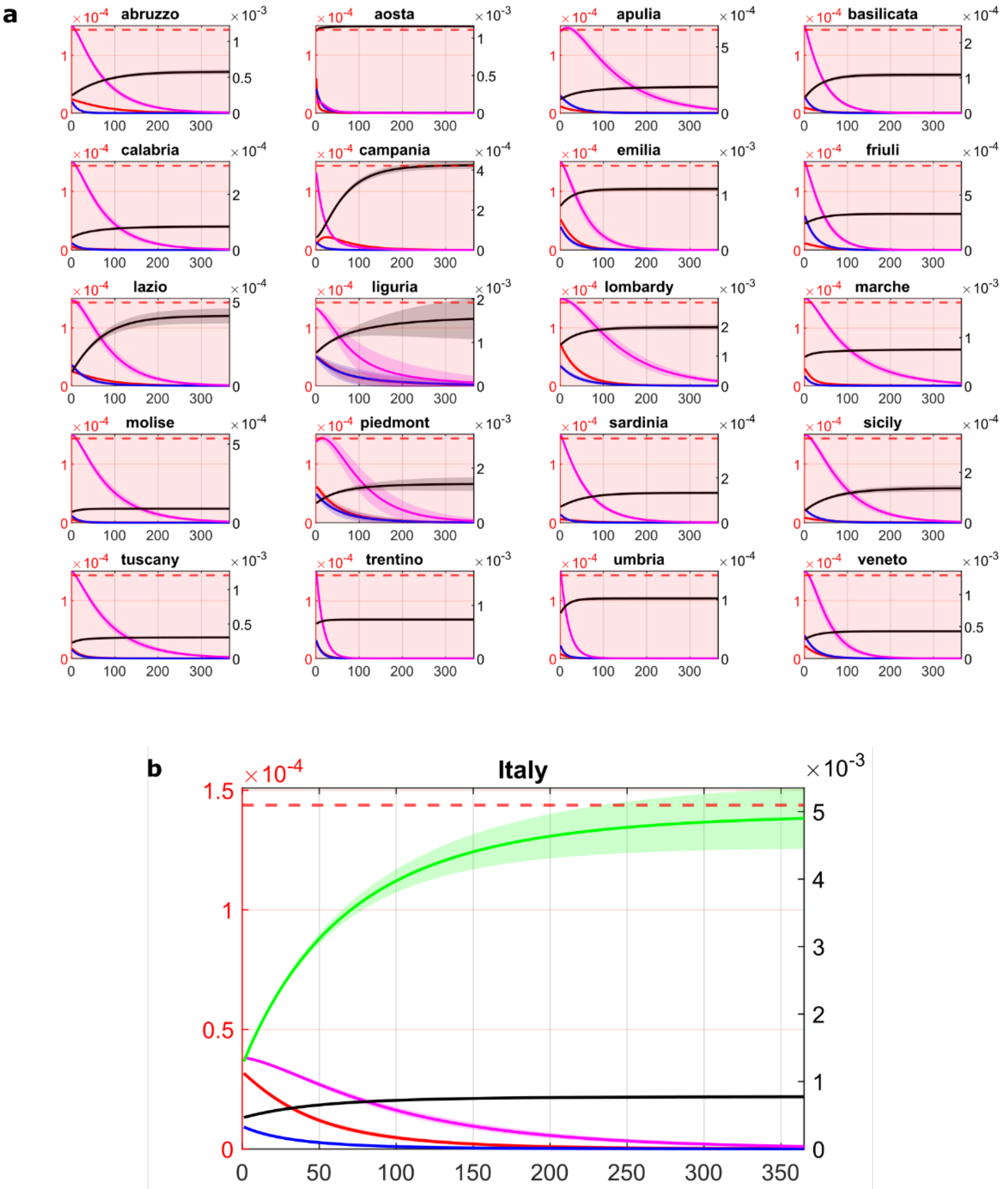

Supplementary Figure 3 [National lockdown] a. Regional and **b.** national dynamics in the case where no region relaxes its containment measures, while all regions restore the interregional fluxes to their pre-lockdown level. Blue, magenta, red, green, and black solid lines correspond to the fraction in the population of infected, quarantined, hospitalized requiring ICU, recovered, and deceased averaged over 10,000 simulations with parameters sampled using a Latin Hypercube technique around their nominal values set as those estimated in the last time window for each region as reported in Supplementary Table 4. Shaded bands correspond to twice the standard deviation. The red dashed line identifies the fraction of the population that can be treated in ICU (T_i^H/N_i). The regions identified with a red label are those where the total hospital capacity is saturated. All plots are shown with a double scale. The scale on the left axis (in red) applies to the hospitalized requiring ICU and the ICU beds capacity threshold, while the right axis (in black) applies to the infected, quarantined, recovered and deceased.

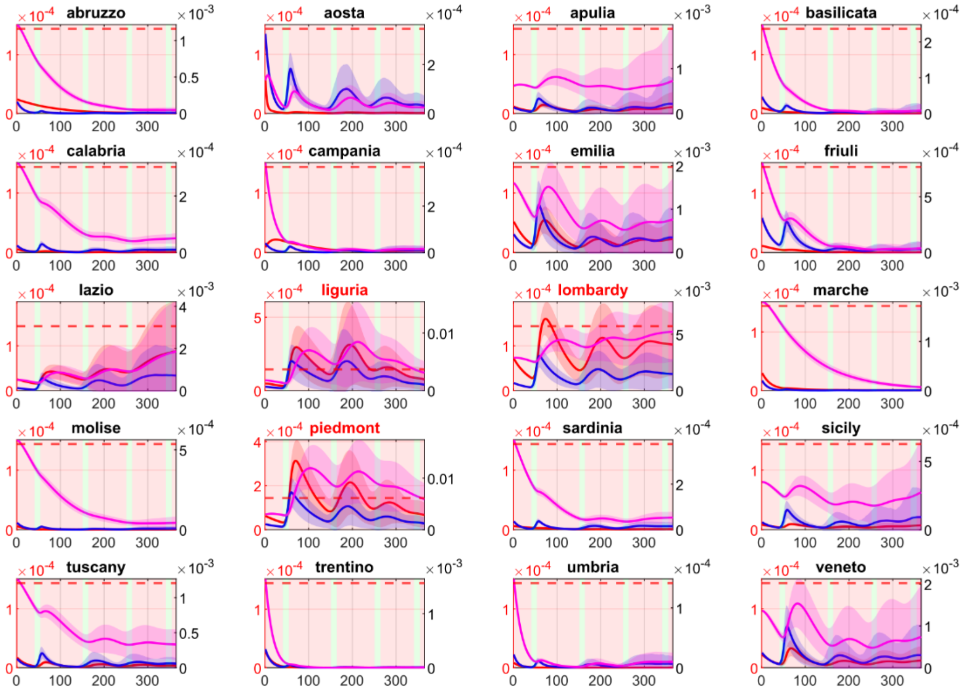

Supplementary Figure 4 [Regional dynamics when intermittent national measures are enforced as shown in Fig. 3c of the main text]. Each of the 20 panels shows the evolution in the corresponding region of the fraction in the population of infected (blue), quarantined (magenta) and hospitalized requiring ICUs (red) averaged over 10,000 simulations with parameters sampled using a Latin Hypercube technique around their nominal values set as those estimated in the last time window for each region as reported in Supplementary Table 4. Shaded bands correspond to twice the standard deviation. The red dashed line identifies the fraction of the population that can be treated in ICU (T_i^H/N_i). National lockdown measures are enforced with all regions shutting down when the total number of occupied ICU beds at the national level exceed 20% (windows shaded in red, green when relaxed). The regions identified with a red label are those where the total hospital capacity is saturated. All plots are shown with a double scale. The scale on the left axis (in red) applies to the hospitalized requiring ICU and the ICU beds capacity threshold, while the right axis (in black) applies to the infected, and quarantined subjects.

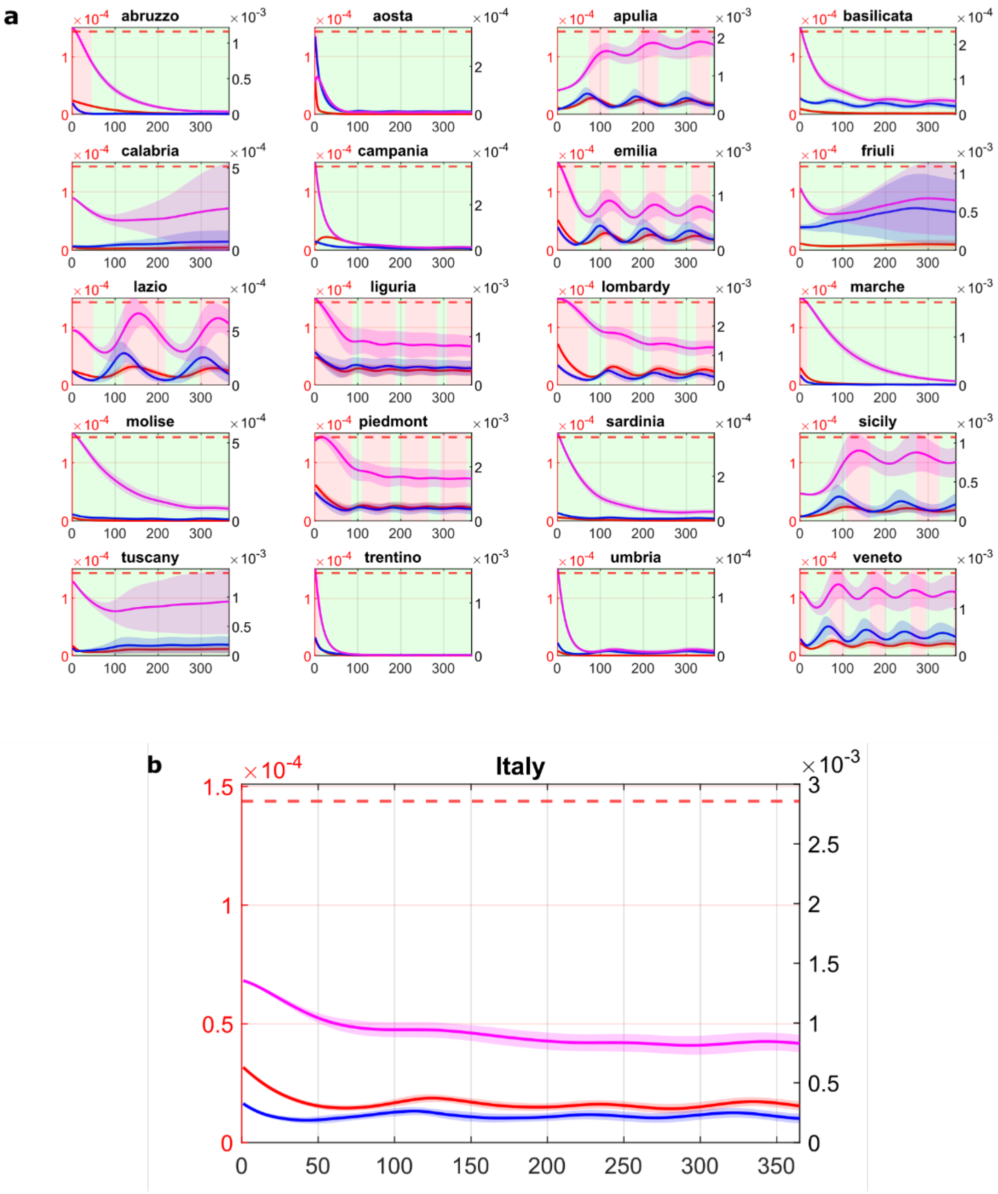

Supplementary Figure 5 [Intermittent regional measures, $\bar{\rho}_i = 1.5\underline{\rho}_i$]. a. Each of the 20 panels shows the evolution in the region named above the panel of the fraction in the population in the population of infected (blue), quarantined (magenta) and hospitalized requiring ICUs (red) averaged over 10,000 simulations with parameters sampled using a Latin Hypercube technique around their nominal values set as those estimated in the last time window for each region as reported in Supplementary Table 4 of the Supplementary Information. Shaded bands correspond to twice the standard deviation. The red dashed line identifies the fraction of the population that can be treated in ICU (T_i^H/N_i). Regions adopt lockdown measures in the time windows shaded in red while relax them in those shaded in green. Differently from Figure 3, when lockdown measures are relaxed $\bar{\rho}_i$ is set to 1.5 times $\underline{\rho}_i$. During a regional lockdown, fluxes in/out of the region are set to their minimum level. **b.** National evolution of the fraction in the population of infected (blue), quarantined (magenta) and hospitalized requiring ICUs (red) obtained by summing those in each of the 20 regions adopting intermittent regional measures. All plots are shown with a double scale. The scale on the left axis (in red) applies to the hospitalized requiring ICU and the ICU beds capacity threshold, while the right axis (in black) applies to the infected and quarantined subjects.

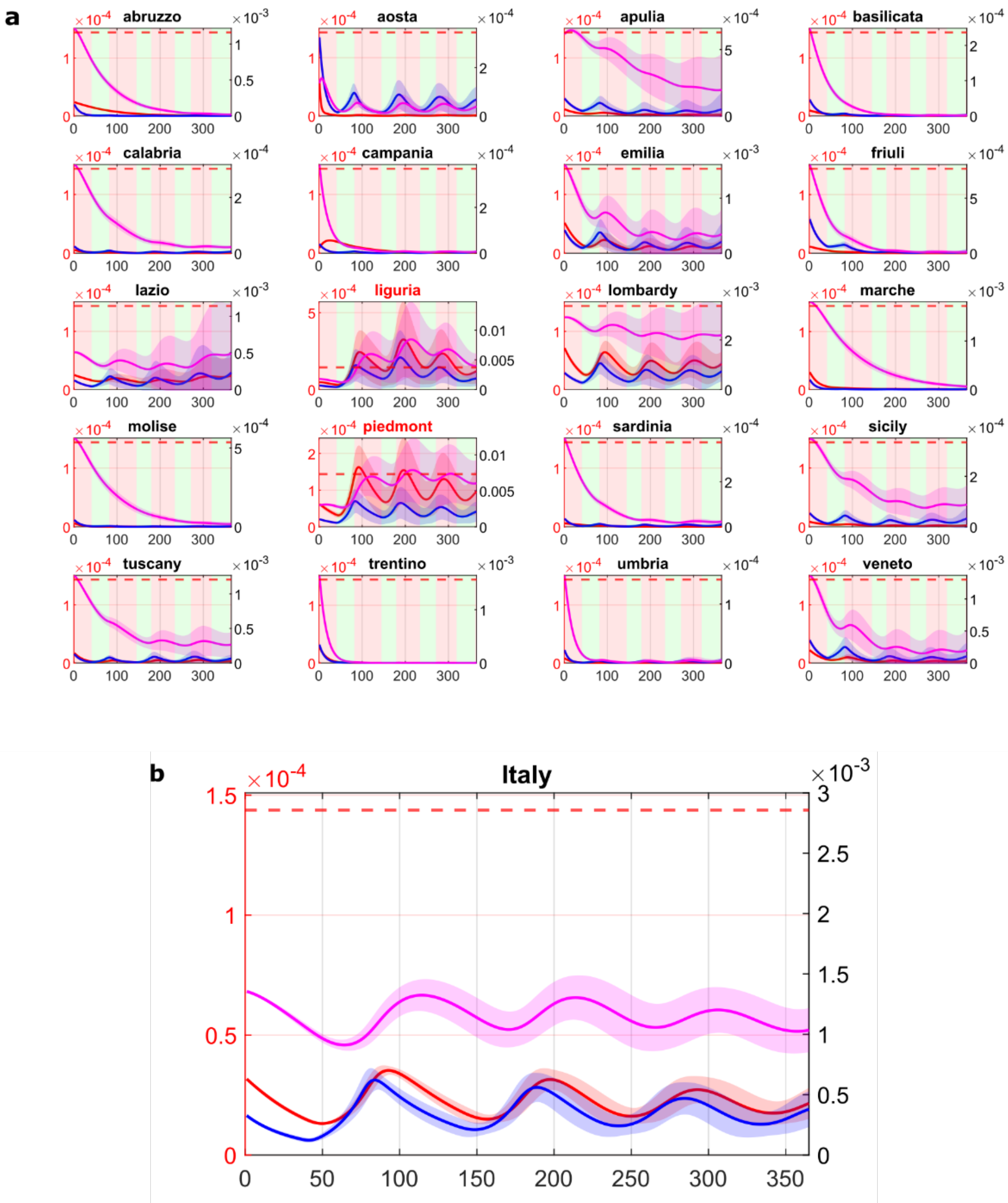

Supplementary Figure 6 [Intermittent national measures, $\bar{\rho}_i = 1.5\underline{\rho_i}$] a. Each of the 20 panels shows the evolution in the corresponding region of the fraction in the population of infected (blue), quarantined (magenta) and hospitalized requiring ICUs (red) averaged over 10,000 simulations with parameters sampled using a Latin Hypercube technique around their nominal values set as those estimated in the last time window for each region as reported in Supplementary Table 4. Shaded bands correspond to twice the standard deviation. The red dashed line identifies the fraction of the population that can be treated in ICU (T_i^H/N_i). National lockdown measures are enforced with all regions shutting down when the total number of occupied ICU beds at the national level exceed 20%. The regions identified with a red label are those where the total hospital capacity is saturated. Regions adopt lockdown measures in the time windows shaded in red while relax them in those shaded in green. Differently from Supplementary Figure 4, when lockdown measures are relaxed $\bar{\rho}_i$ is set to 1.5 times $\underline{\rho}_i$. During a regional lockdown, fluxes in/out of the region are set to their minimum level. **b.** National evolution of the fraction in the population of infected (blue), quarantined (magenta) and hospitalized requiring ICUs (red) obtained by summing those in each of the 20 regions. All plots are shown with a double scale. The scale on the left axis (in red) applies to the hospitalized requiring ICU and the ICU beds capacity threshold, while the right axis (in black) applies to the infected and quarantined subjects.

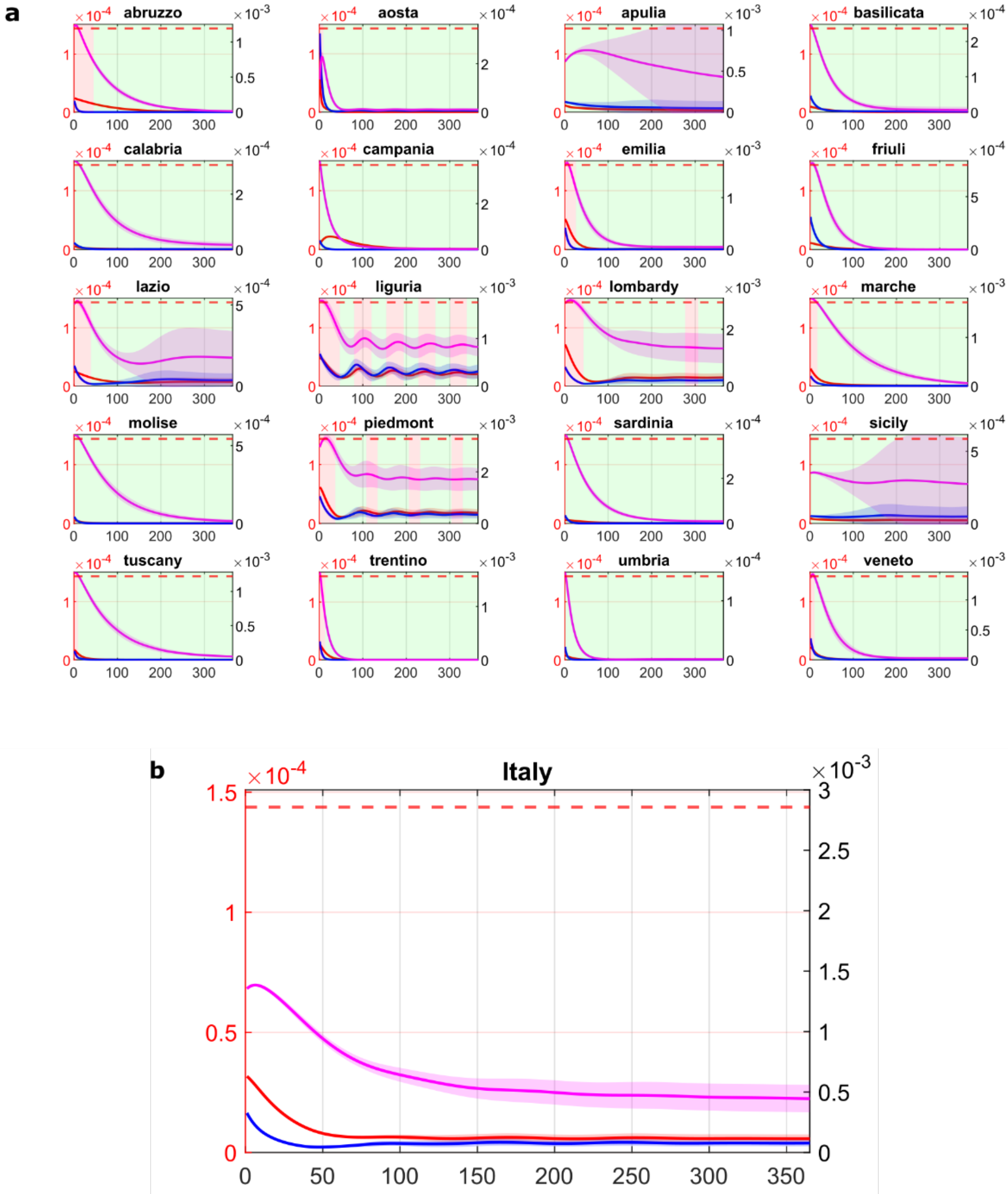

Supplementary Figure 7 [Intermittent regional measures with increased COVID-19 testing capacity and $\bar{\rho}_i = 1.5\underline{\rho}_i$] a. Each of the 20 panels shows the evolution in the region named above the panel of the fraction in the population of infected (blue), quarantined (magenta) and hospitalized requiring ICUs (red) averaged over 10,000 simulations with parameters sampled using a Latin Hypercube technique (see Methods) around their nominal values set as those estimated in the last time window for each region as reported in Supplementary Table 4. Shaded bands correspond to twice the standard deviation. The red dashed line identifies the fraction of the population that can be treated in ICU (T_i^H/N_i). Regions adopt lockdown measures in the time windows shaded in red while relax them in those shaded in green. During a regional lockdown, fluxes in/out of the region are set to their minimum level. Regions COVID-19 testing capacities are assumed to be increased by a factor 2.5 (see Methods) with respect to their current values. Differently from Figure 4, when lockdown measures are relaxed $\bar{\rho}_i$ is set to 1.5 times $\underline{\rho}_i$. During a regional lockdown, fluxes in/out of the region are set to their minimum level. **b.** National evolution of the fraction in the population of infected (blue), quarantined (magenta) and hospitalized requiring ICUs (red) obtained by summing those in each of the 20 regions. All plots are shown with a double scale. The scale on the left axis (in red) applies to the hospitalized requiring ICU and the ICU beds capacity threshold, while the right axis (in black) applies to the infected and quarantined subjects.

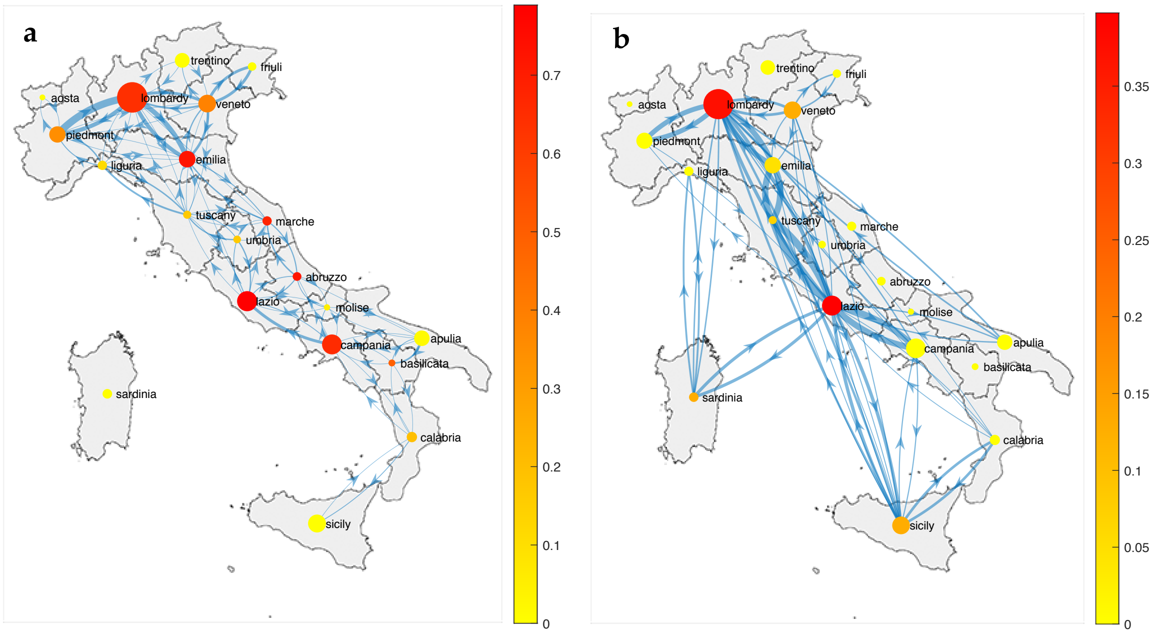

Supplementary Figure 8 [Networks resulting from the estimation of flows] (a) Daily commuters' network; (b) Network topology resulting from the estimation of flows due to high-speed trains, planes, and ferry connections. For the sake of clarity, edges with neglible fluxes are not shown in the figure. Node colours are a measure of their betweenness centrality.

a. **b.**

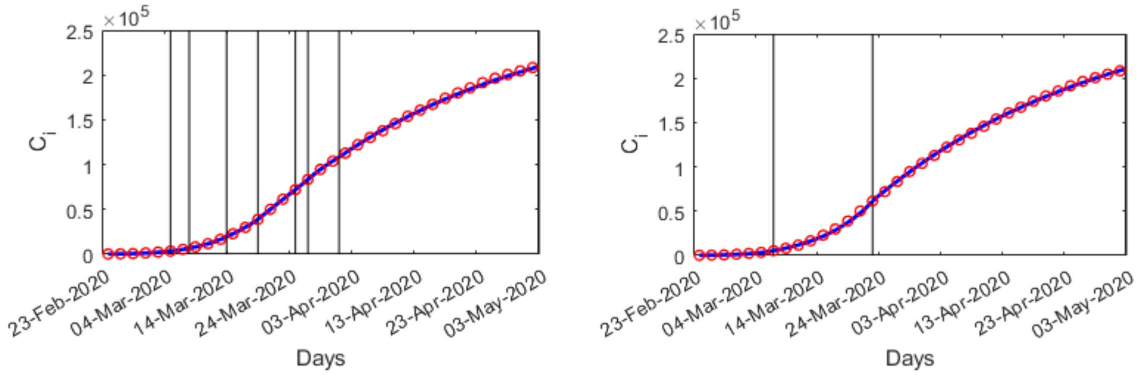

Supplementary Figure 9 [Identification of the aggregate national model] Panel (a). Comparison between model estimates and data collected with time widows identified at the end of Step 1 of the parameter identification process. Panel (b). Comparison between model estimates and data collected with the merged time widows obtained after step 2. In both panels the estimated number of cases estimated by the model $\hat{C}$ (blue solid line) is compared with the available datapoints $\tilde{C}$ (shown as red circles).

a. **b.**

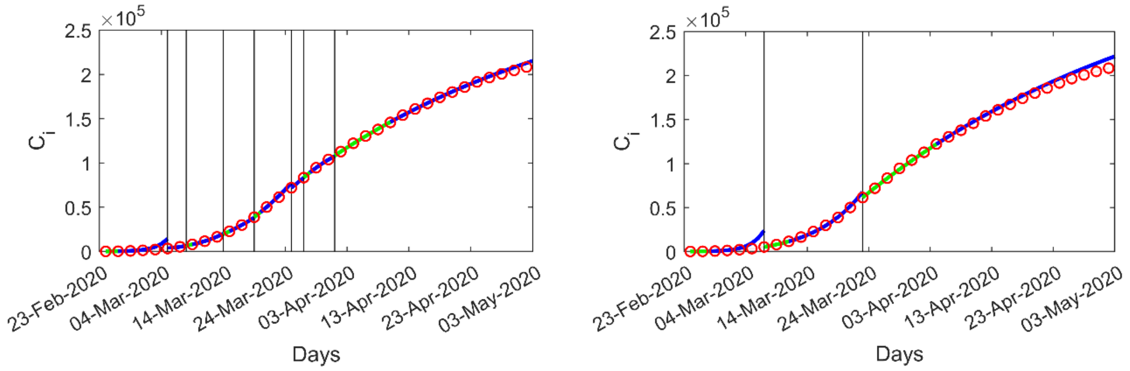

Supplementary Figure 10. [Example of data matching and prediction ability]. Model prediction of the total number of detected cases C_i (blue solid line) at the national level when the parameters are estimated using 30% (green solid line) of all the available data (red circles) in each time window. As shown in panel (a) at the end of step 1, the model estimates match well all the data point in each window. Panel (b) shows the ability of the model to capture the data after the windows are merged as a result of Step 2 of the parameter identification process. The total average mismatch between model estimates and data is less than 10% (and is reduced to less than 1% in the last window).

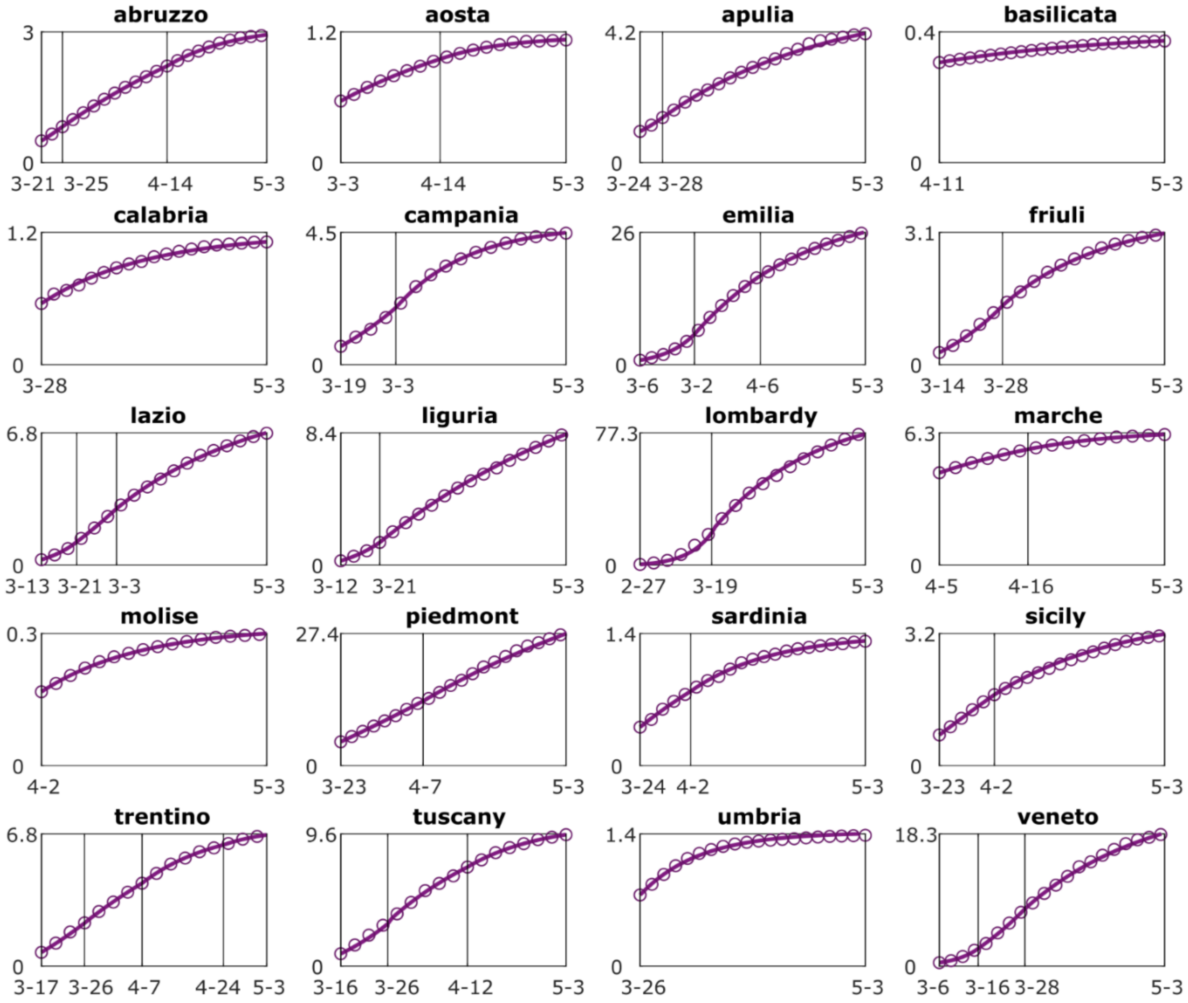

Supplementary Figure 11 [Identification of the regional models - Steps 1,2] Comparison of each of the regional model predictions for the total number (expressed in thousands of people) of detected cases in each region (solid magenta line) against the available data points. Parameters are set to the values estimated at the end of Steps 2 carried out for each region. Vertical black lines denote the breakpoints from one estimation window to the next.

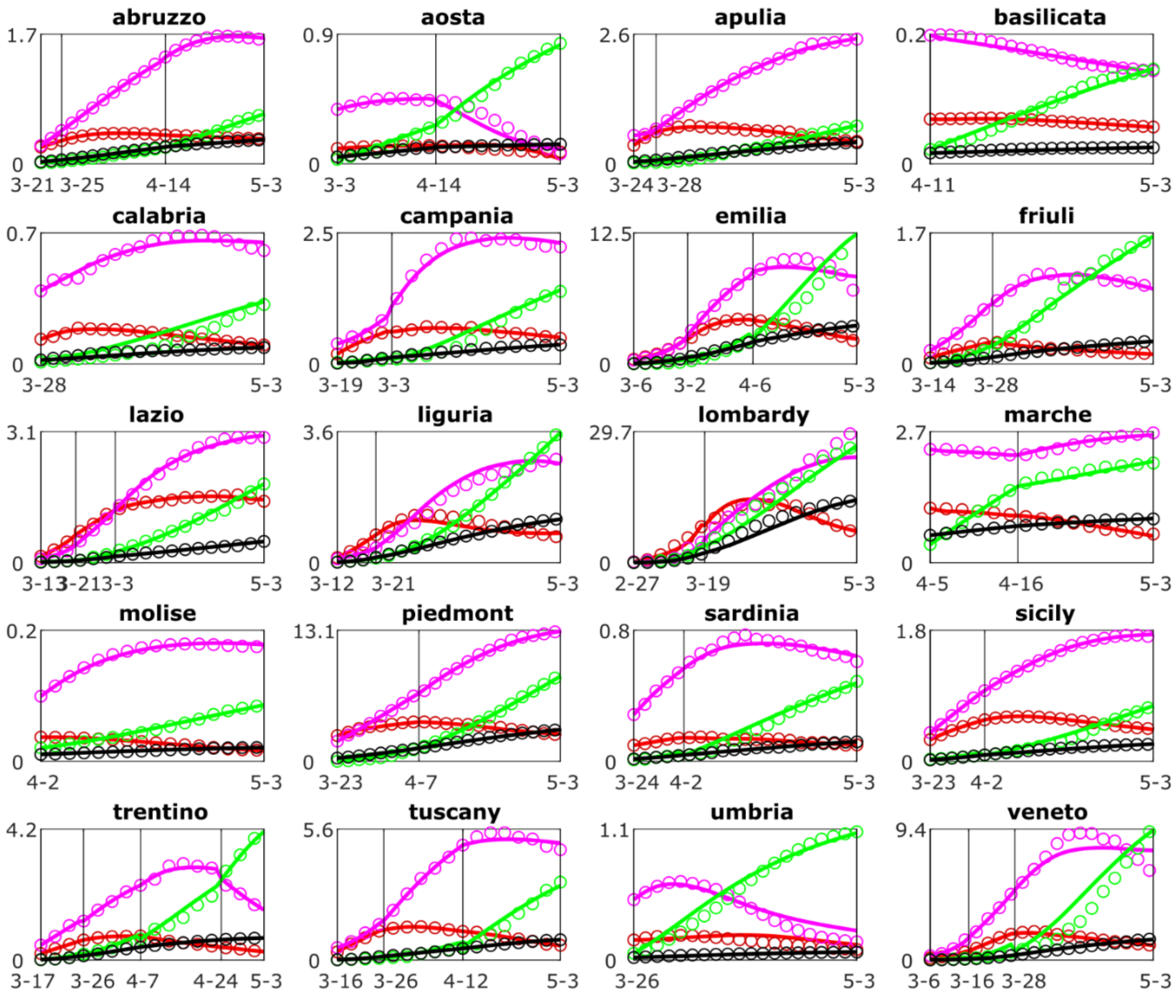

Supplementary Figure 12 [Identification of the regional models - Step 3] Comparison of each of the regional model predictions of the total number (expressed in thousands of people) of recovered (green), quarantined (magenta), hospitalized (red), deceased (black) and recovered (green) in each region against the available data points (plotted as circles of the same colour). Parameters are set to the values estimated at the end of Step 3 carried out for each region. Vertical black lines denote the breakpoints from one estimation window to the next.

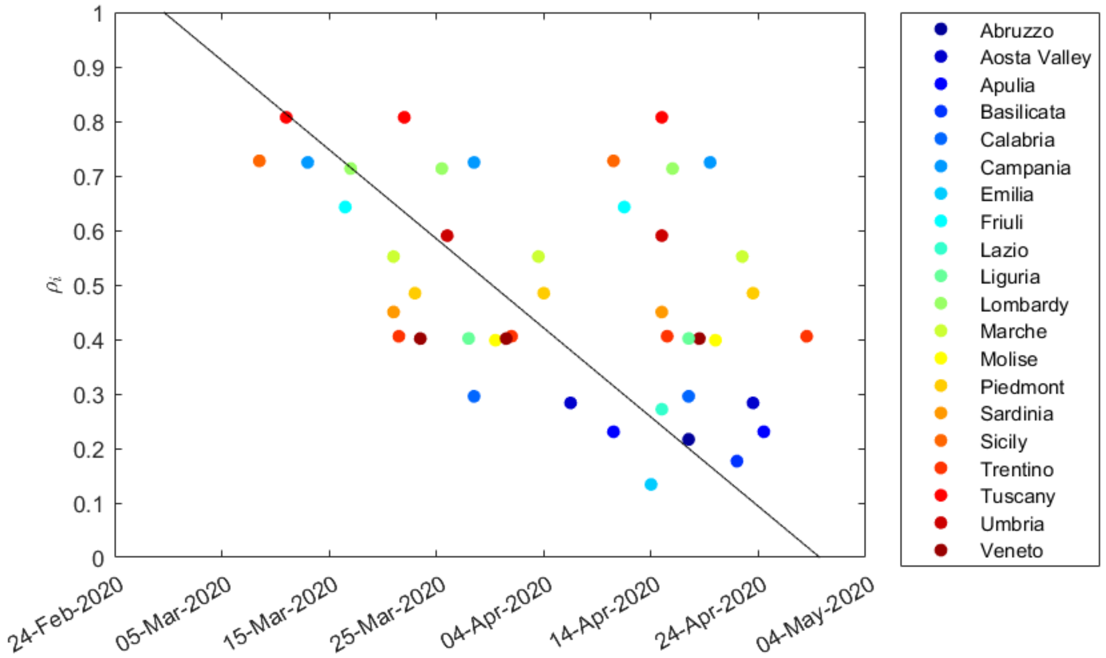

Supplementary Figure 13 [Distribution of the social distancing parameter over time] Distribution of the social distancing parameter ρ_i in the different detected estimation windows. The black line is the LS-interpolant of the National data reported in Supplementary Table 4

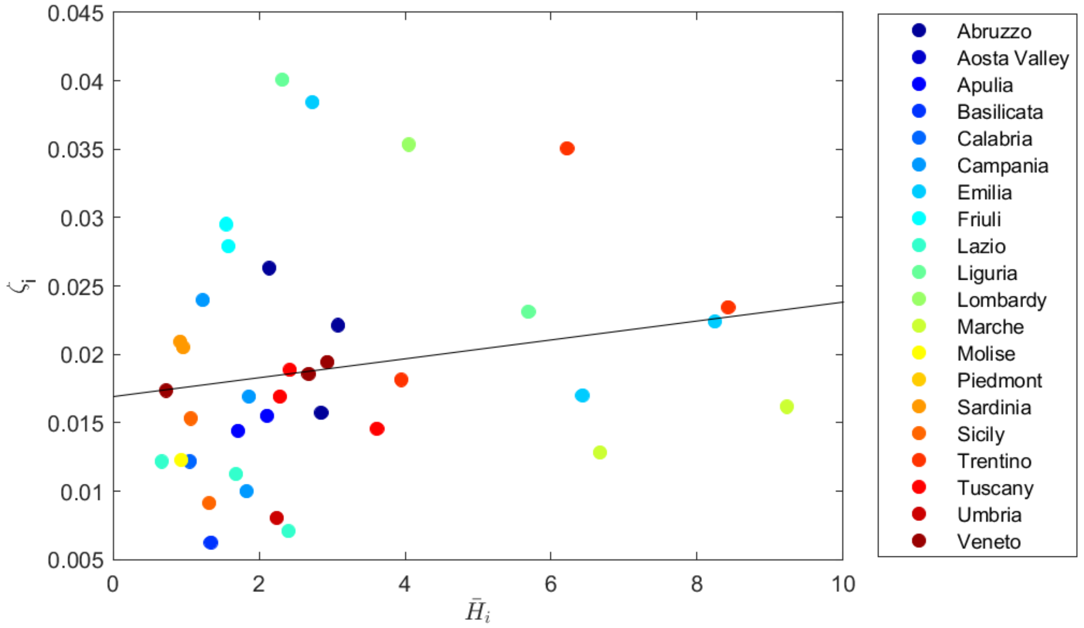

Supplementary Figure 14. [Fitting of ζ_i as a function of $\bar{H}_i$] Each point is the value of ζ_i estimated for a given region in each time window which is plotted against the average number of hospitalized in each time window over the total number of ICU beds available in the same time window. Here, $R^2 = 0.07$, $p = 0.07$, 45 observations, and 43 DOF. Normality of the residuals has been tested with the Lilliefors test (p-value 0.21). The function is saturated at both ends, i.e. at 0 and at the value 0.023 for $\bar{H}_i \geq 10$.

Simulation scenario	Total cases	Total deaths	Maximum hospitalized	Days over hospital's capacity (nation)	Regions over hospital's capacity	Economic cost [M€]
All regions but Lombardy are locked down, Figure 2	10,550,000 ± 146,084	1,196,063 ± 97,122	137,640 ± 10,249	75.8 ± 2.7	3	503,355 ± 0
Intermittent regional measures, Figure 3a,b	1,986,601 ± 76,184	173,637 ± 3,911	2,801 ± 170	0 ± 0	0	509,142 ± 6,606
Intermittent national measure, Figure 3c, Supplementary Figure 4	2,162,539 ± 194,929	205,261 ± 10,854	4,481 ± 277	0 ± 0	3	562,373 ± 12,809
Intermittent regional measures with increased testing, Figure 4	1,590,459 ± 69,118	128,644 ± 2,690	2,057 ± 102	0 ± 0	0	366,514 ± 12,258
All regions but Lombardy are locked down (pre-lockdown fluxes), Supplementary Figure 1	11,655,380 ± 208,245	1,296,978 ± 95,931	140,615 ± 9,976	82.7 ± 2.6	6	503,355 ± 0
No measure is taken, Supplementary Figure 2	54,415,220 ± 218,518	7,278,955 ± 209,729	455,541 ± 15,956	239.4 ± 4.2	20	0 ± 0
National lockdown, Supplementary Figure 3	345,042 ± 29,835	46,756 ± 1,904	1,914 ± 0	0 ± 0	0	643,413 ± 0
Intermittent regional measures $\bar{\rho}_i = 1.5\underline{\rho}_i$ Supplementary Figure 5	995,342 ± 53,097	94,264 ± 2,428	1,914 ± 0	0 ± 0	0	259,314 ± 20,572
Intermittent national measure $\bar{\rho}_i = 1.5\underline{\rho}_i$ Supplementary Figure 6	1,382,216 ± 134,667	128,732 ± 5,591	2,167 ± 84	0 ± 0	2	399,559 ± 43,564
Intermittent regional measures with increased testing and $\bar{\rho}_i = 1.5\underline{\rho}_i$, Supplementary Figure 7	535,668 ± 58,631	57,809 ± 3,899	1,914 ± 0	0 ± 0	0	75,043 ± 16,196

Supplementary Table 1 [Comparison of each of the simulated scenarios over 1 year]. Metrics to evaluate the impact over 1 year of each of the simulated scenarios are reported showing the effectiveness of the Intermittent regional measures in avoiding any saturation of the regional health systems while mitigating the impact of the epidemic and reducing the costs.

Constraint	Description
$0.9\hat{I}^j \leq \hat{I}^{j+1} \leq 1.1\hat{I}^j$;	Continuity constraint on the number of infected at the national level from window j to window $j+1$.
$LB \leq \hat{I}_i^{j+1} \leq UB$<br>$LB = 0.9\hat{I}_i^j - 0.1\hat{I}^j$<br>$UB = 1.1\hat{I}_i^j + 0.1\hat{I}^j$	Continuity constraint on the number of infected at the regional level. The constraints are relaxed by 10% of the national estimated infected to account for the fact that in estimating the region parameter we are neglecting the influx of infected from other regions.
$\hat{\kappa}^Q \leq 0.1$,<br>$\hat{\kappa}^H \leq 0.1$;	We assume that the daily number of people hospitalized from quarantine and discharged but still positive (and vice versa) is no higher than 10% of the total.
$0.7\tau^j \leq \alpha^j + \psi^j \leq 1.3\tau^j$	We assume $\hat{\tau}_i = \hat{\alpha}_i + \hat{\psi}_i$ does not differ from the national estimate $\hat{\tau}$ of more than 30%.
$\eta^{Q,j+1} = \eta^{Q,j}$	We assume the recovery rate of those quarantined at home remains the same from a time window to the next as this parameter is likely to be time-invariant. In any case, removing this constraint, we observed no significant change of this parameter from a time window to the next.

Supplementary Table 2 [Constraints for the regional models' parameterization]. Set of parameters constraints enforced by the parameter identification algorithm.

	ρ	I_0	I_f	η_Q	η_H	ζ	α	ψ	κ_H	κ_Q	t_i	$R_{0,i}$
						Step 1						
*	0.965	1200	11580	0.028	0.045	0.023	0.020	0.050	0.000	0.000	24-feb-20	2.76
*	0.958	11580	18805	0.028	0.013	0.024	0.018	0.059	0.027	0.049	05-mar-20	2.61
†	0.774	18805	43814	0.028	0.017	0.029	0.034	0.042	0.000	0.000	08-mar-20	2.11
†	0.607	43814	65971	0.028	0.019	0.029	0.048	0.025	0.100	0.000	14-mar-20	1.70
†	0.331	65971	56726	0.028	0.008	0.032	0.053	0.037	0.099	0.100	19-mar-20	0.83
‡	0.533	56726	59229	0.028	0.002	0.029	0.061	0.042	0.045	0.054	25-mar-20	1.24
‡	0.234	59229	44109	0.028	0.000	0.027	0.049	0.043	0.056	0.100	27-mar-20	0.58
‡	0.374	44109	17980	0.028	0.000	0.018	0.023	0.085	0.001	0.088	01-apr-20	0.84
						Step 2						
*	0.937	1200	16639	0.028	0.029	0.022	0.018	0.053	0.000	0.000	24-feb-20	2.64
†	0.646	16639	78621	0.028	0.014	0.032	0.018	0.074	0.000	0.100	07-mar-20	1.59
‡	0.298	78621	26392	0.028	0.000	0.020	0.019	0.055	0.000	0.080	23-mar-20	0.82

Supplementary Table 3 [Parameters of the aggregate national model] Parameters of the aggregate national model before and after Step 2. Parameters values are given before and after merging the time windows. Symbols at the beginning of each row denote parameters from windows that are then merged in Step 2 of the identification procedure. Note that because of the nonlinear nature of the model, parameter values in the merged windows (after Step 2) can exceed the ranges of the separate windows obtained in Step 1.

Region	ρ	I_0	I_f	η_Q	η_H	ζ	α	ψ	κ_H	κ_Q	t_i	R_0
Abruzzo	0,485	944	1083	0,010	0,003	0,026	0,029	0,051	0,005	0,099	21-mar-20	1,29
	0,321	1083	747	0,010	0,000	0,022	0,025	0,049	0,000	0,087	25-mar-20	0,89
	0,194	847	210	0,010	0,019	0,014	0,078	0,003	0,005	0,000	14-apr-20	0,51
Aosta	0,283	425	262	0,010	0,096	0,036	0,062	0,016	0,028	0,000	30-mar-20	0,77
	0,122	262	41	0,010	0,260	0,011	0,062	0,000	0,079	0,000	14-apr-20	0,37
Apulia	0,590	1300	1732	0,010	0,000	0,016	0,028	0,047	0,100	0,100	24-mar-20	1,62
	0,278	1732	530	0,010	0,004	0,015	0,022	0,051	0,002	0,078	28-mar-20	0,78
Basilicata	0,177	90	26	0,010	0,060	0,006	0,037	0,021	0,025	0,025	11-apr-20	0,55
Calabria	0,272	384	49	0,015	0,000	0,012	0,042	0,059	0,005	0,079	28-mar-20	0,63
Campania	0,467	1231	1816	0,018	0,000	0,022	0,014	0,064	0,000	0,100	19-mar-20	1,26
	0,221	2231	234	0,018	0,000	0,011	0,067	0,019	0,006	0,040	30-mar-20	0,57
Emilia	0,725	1418	7246	0,029	0,000	0,038	0,020	0,089	0,000	0,100	06-mar-20	1,62
	0,400	7246	4467	0,029	0,000	0,023	0,059	0,062	0,000	0,045	20-mar-20	0,84
	0,362	4467	1881	0,029	0,031	0,017	0,063	0,050	0,000	0,017	06-apr-20	0,79
Friuli	0,450	900	1717	0,028	0,022	0,028	0,032	0,034	0,000	0,100	14-mar-20	1,32
	0,202	1717	376	0,028	0,049	0,029	0,044	0,007	0,004	0,000	28-mar-20	0,67
Lazio	0,713	722	1689	0,015	0,003	0,013	0,018	0,081	0,000	0,062	13-mar-20	1,69
	0,483	1689	1995	0,015	0,012	0,012	0,029	0,076	0,055	0,100	21-mar-20	1,11
	0,330	1995	732	0,015	0,008	0,007	0,024	0,066	0,039	0,100	30-mar-20	0,82
Liguria	0,643	900	1933	0,037	0,010	0,040	0,030	0,070	0,100	0,062	12-mar-20	1,52
	0,398	2126	1053	0,037	0,010	0,023	0,012	0,092	0,000	0,100	21-mar-20	0,92
Lombardy	0,727	1799	28900	0,010	0,053	0,033	0,009	0,092	0,000	0,040	27-feb-20	1,69
	0,303	28900	6731	0,010	0,029	0,024	0,018	0,056	0,000	0,027	19-mar-20	0,84
Marche	0,231	1906	1206	0,010	0,080	0,016	0,022	0,047	0,009	0,000	05-apr-20	0,66
	0,133	1178	311	0,010	0,007	0,011	0,000	0,057	0,002	0,068	16-apr-20	0,42
Molise	0,217	120	13	0,013	0,000	0,012	0,067	0,018	0,000	0,043	02-apr-20	0,56
Piedmont	0,398	6527	7244	0,022	0,000	0,019	0,010	0,073	0,000	0,100	23-mar-20	1,05
	0,363	7244	4588	0,022	0,014	0,021	0,021	0,071	0,000	0,100	07-apr-20	0,90
Sardinia	0,296	618	487	0,013	0,000	0,022	0,064	0,017	0,026	0,100	24-mar-20	0,78
	0,216	487	60	0,013	0,038	0,021	0,066	0,017	0,015	0,063	02-apr-20	0,56
Sicily	0,402	1025	952	0,015	0,000	0,016	0,048	0,055	0,034	0,100	23-mar-20	0,93
	0,293	952	271	0,015	0,000	0,009	0,017	0,068	0,012	0,100	02-apr-20	0,75
Trentino	0,406	2305	2867	0,029	0,000	0,035	0,048	0,024	0,001	0,000	17-mar-20	1,14
	0,291	2867	2261	0,029	0,000	0,032	0,032	0,038	0,004	0,100	26-mar-20	0,83
	0,226	2261	802	0,029	0,035	0,023	0,081	0,006	0,002	0,000	07-apr-20	0,58
	0,201	802	368	0,029	0,320	0,018	0,073	0,017	0,060	0,100	24-apr-20	0,50
Tuscany	0,552	1675	2666	0,012	0,000	0,017	0,031	0,086	0,014	0,100	16-mar-20	1,18
	0,353	2932	1690	0,012	0,000	0,014	0,046	0,062	0,000	0,093	26-mar-20	0,79
	0,317	1690	482	0,012	0,064	0,019	0,063	0,055	0,001	0,008	12-apr-20	0,68
Umbria	0,134	794	19	0,010	0,141	0,008	0,089	0,000	0,052	0,000	26-mar-20	0,34
Veneto	0,807	848	3538	0,031	0,000	0,018	0,062	0,052	0,000	0,100	06-mar-20	1,75
	0,520	3538	5089	0,031	0,000	0,019	0,078	0,044	0,000	0,039	16-mar-20	1,08
	0,336	5089	1749	0,031	0,000	0,019	0,054	0,048	0,002	0,100	28-mar-20	0,78

Supplementary Table 4 [Model Parameters] Values of estimated parameters for each region at the end of the identification process. Dates are given corresponding to breakpoints between estimation windows that are automatically detected by the estimation procedure we proposed. Regional net reproduction number are reported in the last column clearly showing the increasing effect of the national lockdown measures taken by the government on March 8th, 2020. The parameter values used to simulate the exit from the Lock-Down considered in this paper are those shaded in grey for each region corresponding to the estimated parameter in the last window ending on May 3rd 2020. Note that for most regions the analysis started from a date when social distancing measures were already in place hence the estimated initial R_0 are lower than the values reported in the literature before those measures were in place (e.g. a value of 3-4 for Lombardy for instance).

Source	Model	ρ			β			$\rho \cdot \beta$ (S → I)			α (I → Q)			γ (I → R)			ψ (I → H)			κ^H (Q → H)			κ^Q (H → Q)			η^Q (Q → R)			η^H (H → R)			ζ (H → D)			R_0		
		nom	min	max	nom	min	max	nom	min	max	nom	min	max	nom	min	max	nom	min	max	nom	min	max	nom	min	max	nom	min	max	nom	min	max	nom	min	max	nom	min	max
our model	SIQHRD	variable	0.122	1	0.4	0.4	0.4	0.114	0.049	0.159	0.044	1E-15	0.089	0.07	0.07	0.07	0.033	8.68E-11	0.092	0.006	1.82E-15	0.079	0.045	8.26E-14	0.1	0.018	0.01	0.037	0.016	1E-19	0.32	0.02	0.006	0.029	0.78	0.195	2.272
[S6]	SEPIAHQRD	-	-	-	0.301	0.273	0.33	-	-	-	0.099	0.093	0.104	0.087	0.077	0.094	0.148	0.13986	0.156	-	-	-	-	-	-	0.07	0.063	0.073	0.07	0.063	0.073	0.041	0.037	0.045	3.6	3.49	3.84
[S3]	SIDARTHE	-	-	-	-	-	-	-	-	-	-	-	-	0.034	-	-	0.017	-	-	0.027	-	-	-	-	-	-	-	-	0.017	-	-	0.01	-	-	-	-	-
[S14]	SIR	-	-	-	-	0.26	0.315	-	-	-	-	-	-	-	-	-	-	-	-	-	-	-	-	-	-	-	-	-	-	-	-	-	-	-	-	2	4
[S15]	SIQR	-	-	-	0.373	-	-	-	-	-	0.067	-	-	-	-	-	-	-	-	-	-	-	-	-	-	-	-	-	-	-	-	-	-	-	-	-	-
[S4]	SIRD	-	-	-	-	-	-	-	0.143	0.348	-	-	-	-	-	-	-	-	-	-	-	-	-	-	-	-	-	-	-	-	-	-	-	-	-	-	-
[S16]	-	-	-	-	-	-	-	-	-	-	-	-	-	-	-	-	-	-	-	-	-	-	-	-	-	-	-	-	-	-	-	-	-	-	-	2	4
Mean, min, max →		-	-	-	0.337	0.26	0.373	-	0.143	0.348	0.083	0.067	0.104	0.061	0.034	0.094	0.083	0.017	0.156	0.027	0.027	0.027	-	-	-	0.07	0.063	0.073	0.043	0.017	0.073	0.026	0.01	0.045	3.6	2	4
Source		Correspondence in source																																			
[S6]		λ			δ_E			-			$\zeta \cdot \eta$			$(\delta_E, \sigma, \delta_P, \gamma_A)$			$(1 - \zeta) \cdot \eta$			-			-			γ_Q			γ_H			α_H			R_0		
[S3]		-			-			$\alpha, \beta, \gamma, \delta$			ε, θ			λ			μ			ν			-			ρ, ξ			σ			τ			-		
[S14]		-			β_0			-			-			-			-			-			-			-			-			-			R_0		
[S15]		-			β			-			η			-			-			-			-			-			-			-			-		
[S4]		-			-			β			-			-			-			-			-			-			-			-			-		
[S16]		-			-			-			-			-			-			-			-			-			-			-			R_0		

Supplementary Table 5 [Comparison with the parameters estimated in other papers in the literature for the national aggregate models]. Comparison between the parameter's values we used in our work and those used in other papers proposing national models for the COVID-19 epidemic in Italy that recently appeared in the literature. Notice that, unfortunately, often it is not possible to pin down a specific parameter in a different model that clearly corresponds to one of ours, and vice-versa. This is because of the different meaning that compartments have in the models and the dynamics of people between the compartments which do not always overlap in an unambiguous manner. When we had to use a time constant, say τ, to determine the value of a parameter, say k, we set $k = 1 / \tau$.
